# Non-proteinogenic β-alanine metabolism in *Halomonas* sp. MNB13 regulates manganese reduction in deep-sea ferromanganese nodules

**DOI:** 10.1128/aem.02164-25

**Published:** 2025-11-18

**Authors:** Shuju Guo, Xiuli Xu, Hui Shuai, Fuhang Song, Guoliang Zhang, Linlin Ma, Na Yang

**Affiliations:** 1 Laboratory of Experimental Marine Biology, Institute of Oceanology, Chinese Academy of Sciences 53014 , Qingdao, P.R. China; 2 Laboratory for Marine Biology and Biotechnology, Qingdao National Laboratory for Marine Science and Technology 474988 , Qingdao, P.R. China; 3 Key Laboratory of Marine Mineral Resources and Polar Geology, Ministry of Education of China, School of Ocean Sciences, China University of Geosciences 113084 , Beijing, P.R. China; 4 State Key Laboratory of Integration and Innovation of Classic Formula and Modern Chinese Medicine, Lunan Pharmaceutical Group Co Ltd 638156 , Linyi, P.R. China; 5 Department of Marine Technology, Rizhao Polytechnic, Rizhao, Shandong, P.R. China; 6 School of Light Industry, Beijing Technology and Business University 58276, Beijing, P.R. China; 7 School of Marine Sciences, Sun Yat-sen University 212170 , Zhuhai, P.R. China; 8 Institute for Biomedicine and Glycomics, School of Environment and Science, Griffith University 5723, Brisbane, Australia; Colorado School of Mines, Golden, Colorado, USA

**Keywords:** Deep-sea ferromanganese nodules, Mn reduction, *Halomonas*sp. MNB13, β-Alanine metabolism, organic acids

## Abstract

Deep-sea ferromanganese nodules are valuable materials for investigating microorganism-mediated geochemical cycling of manganese. While many microbial metabolic pathways are closely associated with manganese reduction, few studies have examined the role of amino acid metabolism in the release of Mn(II) from these nodules. Here, we explored the impact of non-proteinogenic β-alanine metabolism on manganese reduction. *Halomonas* sp. MNB13 is an indigenous bacterium isolated from a ferromanganese nodule that can reduce Mn oxides and decrease the pH of the environment during Mn(II) release. Comparative transcriptomic analysis unveiled that exposure to MnO_2_ significantly upregulated β-alanine metabolism, leading to a reduced intracellular concentration of β-alanine. Exogenous β-alanine decreased the proportion of Mn(IV) without changing the type of Mn residues by promoting MNB13 growth and reshaping the profile of secreted organic acids. β-Alanine elevated energy metabolism in MNB13 and increased catalase activity, mitigating reactive oxygen species generated during Mn(II) release and thereby enhancing bacterial growth. Among the secreted organic acids, levulinate and pantothenate showed altered abundance and were confirmed to promote Mn(II) release following β-alanine supplementation. This study suggests that environmental β-alanine modulates Mn(IV) reduction by promoting bacterial growth and organic acid secretion, offering novel insights into bacteria-mediated Mn(II) release from deep-sea ferromanganese nodules.

**IMPORTANCE:**

Microorganisms are believed to play a role in the biotic processes of deep-sea ferromanganese nodule formation and biosolubilization. Although most studies have linked microbial metabolism to manganese reduction, the role of microbial amino acid metabolism in the release of Mn(Ⅱ) from ferromanganese nodules into seawater remains underexplored. β-Alanine is a naturally occurring non-proteinogenic β-amino acid widely distributed in the marine environment. However, its role in Mn(Ⅳ) reduction is unclear. This study demonstrated that Mn oxides upregulate β-alanine metabolism in *Halomonas* sp. MNB13 and external β-alanine significantly enhances Mn(Ⅱ) release from Mn oxides. We showed that ferromanganese nodules induce β-alanine metabolism and lead to the release of pantothenate and levulinate, decreasing the culture pH, reducing Mn(Ⅳ) to Mn(Ⅱ), and resulting in Mn(Ⅱ) release. These findings provide new insights into bacterial non-proteinogenic amino acid metabolism and its role in facilitating the Mn(Ⅱ) release from ferromanganese nodules in deep-sea environments.

## INTRODUCTION

Deep-sea ferromanganese nodules cover vast areas of the ocean floor and represent a unique abyssal habitat (1). Since their discovery, numerous studies have investigated microbial interactions with these nodules, revealing their critical role in geochemical cycling of element manganese (Mn) (1–3). These nodules harbor diverse prokaryotic communities dominated by Mn(IV)-reducing and Mn(II)-oxidizing bacteria (4), in which microorganisms facilitate the solubilization of particulate Mn oxides and subsequent metal release via reduction of high-valent Mn(III/IV) (5). Although specific strains such as *Jeotgalibacillus campisalis* are implicated in Mn oxide dissolution (6), the metabolic mechanisms governing microbial nodule decomposition remain incompletely understood.

Microbial Mn reduction occurs in anoxic or oxic conditions through direct enzyme action (e.g., Mn reductase) or indirectly via metabolic byproducts functioning as reducing agents (7–14). *Shewanella* spp. and *Geobacter* spp. are key taxa that instigate extracellular electron transfer to reduce Mn oxides as terminal electron acceptors during anaerobic respiration (13, 15–17). Alternatively, certain microorganisms secrete extracellular polymeric substances, enzymes, and metabolites to facilitate Mn dissolution. For example, siderophores dissolve Mn oxides by forming siderophore-Mn(III) complexes (18), while humic acids and quinone-containing compounds act as electron shuttles to reduce Mn oxides (14, 19). Meanwhile, sulfur compounds, such as thiosulfate or sulfide, contribute to Mn reduction as electron donors (20). Similarly, organic acids produced by fermenting bacteria can act as electron donors and reduce environmental pH, thereby promoting the dissolution of Mn oxides in ferromanganese nodules (5, 21–23). Despite these advances, the influence of bacterial amino acid metabolism on Mn reduction remains unexplored.

The role of β-alanine in Mn reduction should be explored since its metabolism can lead to the production of organic acids. In marine ecosystems, β-alanine primarily arises from bacterial decarboxylation of aspartate and glutamate, serving as a precursor for coenzyme A synthesis and other metabolites (24). Research on bacterial β-alanine has primarily focused on its biosynthetic and metabolic pathways to facilitate large-scale production (25–27). Nevertheless, the role of β-alanine in Mn cycling, particularly in microorganism-mediated release of Mn(II) from ferromanganese nodules, remains largely unexplored.

We previously isolated *Halomonas* sp. MNB13 from a deep-sea ferromanganese nodule collected in the Western Pacific Ocean. This strain has Mn(II)-oxidizing activity linked to the modulation of cystine metabolism (28). In this study, we hypothesize that β-alanine metabolism in *Halomonas* sp. MNB13 is involved in Mn(IV) reduction and Mn(II) release from ferromanganese nodules. To test this hypothesis, we combined transcriptomics, enzyme activity assays, and measurements of secreted organic acids to elucidate the underlying biochemical mechanism.

## RESULTS AND DISCUSSION

### Mn reduction by *Halomonas* sp. MNB13

**Fig 1:**
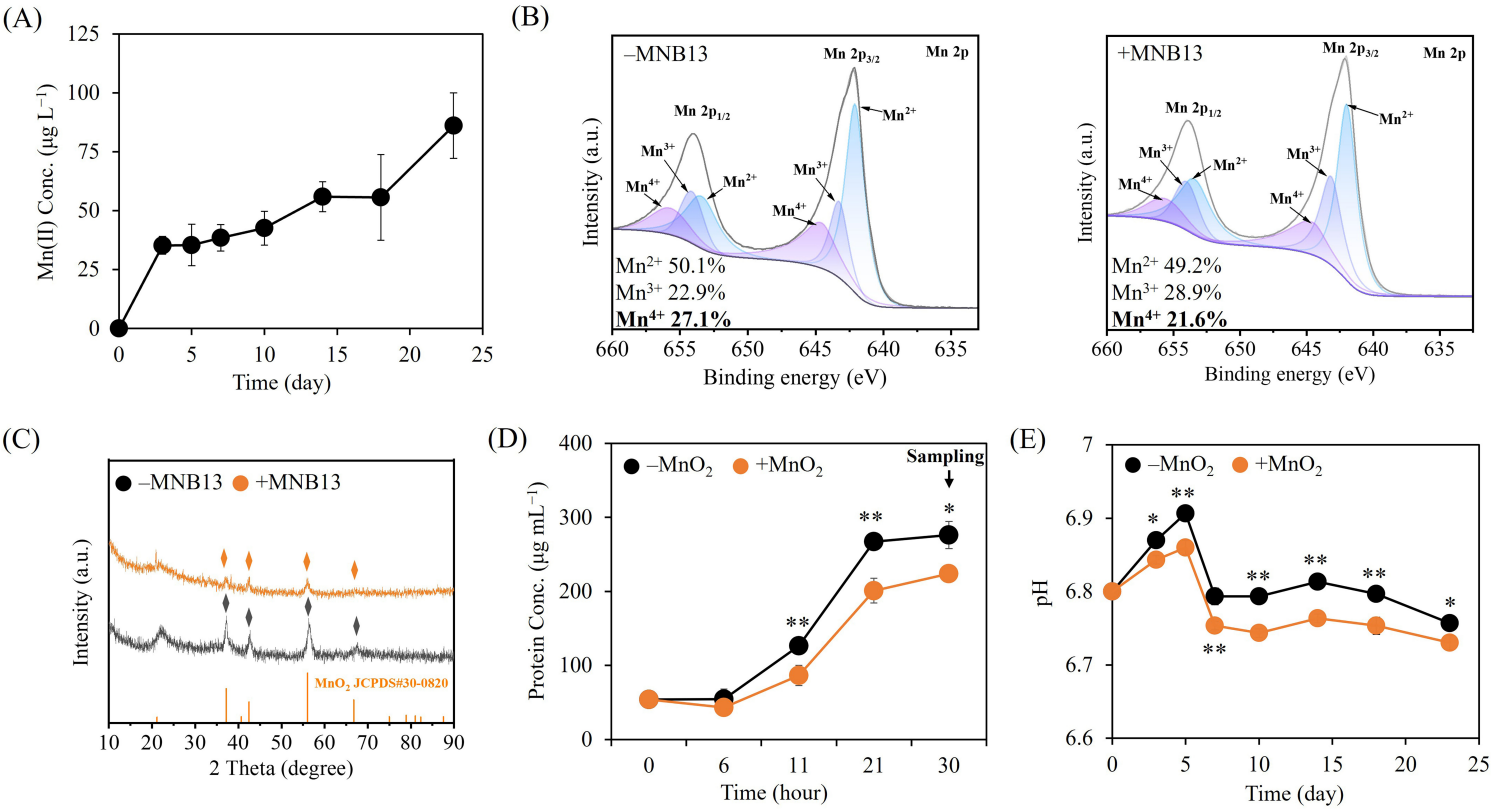
Performance of *Halomonas* sp. MNB13 in basal medium with 0.5% MnO_2_ (wt/vol) under aerobic conditions. (**A**) Aqueous Mn(II) concentration. (**B**) Mn (2p) spectrograms of Mn residues after 28 days of cultivation. (**C**) X-ray diffraction patterns of Mn residues after 28 days of cultivation. (**D**) Growth curves of strain MNB13 with and without MnO_2_ supplementation. The arrow indicates the time points at which cultures were sampled for transcriptomic analysis. (**E**) Culture pH changes during MnO_2_ reduction. *P < 0.05; **P < 0.01.

Given that deep-sea polymetallic nodules serve as an oxygen resource in abyssal seafloor environments (29, 30), all MnO_2_ reduction assays with strain MNB13 were performed under oxic conditions. Strain MNB13 caused a rapid increase in Mn(II) release during the first three days (from 0 to 35.3 µg L^−1^), after which the rate declined (Fig. 1A), probably due to re-adsorption of dissolved Mn(II) onto cell surface or residual Mn oxides (31, 32). We further quantified the valence-state distribution of Mn in cultured residues to validate the MnO_2_-reducing capability of strain MNB13. X-ray photoelectron spectroscopy (XPS) demonstrated a decrease in the proportion of Mn(IV) from 27.1% to 21.6%, accompanied by an increase in Mn(III) from 22.9% to 28.9% after inoculation with strain MNB13 (Fig. 1B), confirming its involvement in MnO_2_ reduction. These findings expand the functional repertoire of *Halomonas*, a genus previously characterized primarily for Mn resistance and Mn(II) oxidation capabilities, rather than Mn reduction (11, 28, 33–36).

X-ray diffraction (XRD) analysis of residuals from MNB13-treated cultures showed identical characteristic peaks corresponding to ε-MnO_2_ (36.12°, 42.40°, 56.03°, and 66.76°; JCPDS#30-0820) but with attenuated intensity relative to the control (Fig. 1C), indicating the absence of secondary Mn mineral phase formation during MnO_2_ reduction. The reduction of Mn mineral birnessite by *Dietzia* sp. DQ12-45-1b coincides with aragonite precipitation, driven by acetate oxidation that elevates cultural pH via increased CO_3_^2-^ concentration (14, 37). In contrast, no secondary Mn minerals were detected during MNB13-mediated MnO_2_ reduction. This may be attributed to the oligotrophic conditions of our reduction medium, which contained only 0.05% (wt/vol) yeast extract as the carbon source, thereby limiting alkalinization and carbonate supersaturation necessary for mineral co-precipitation.

A positive correlation between aerobic Mn(IV) reduction and bacterial growth has been reported (14). To access whether MnO_2_ affects the growth of strain MNB13, we measured the whole-cell protein concentration in culture. MnO_2_ supplementation resulted in significantly decreased growth (*P* < 0.05) starting from the mid-exponential phase and led to a markedly lower cell density (*P* < 0.05) during the stationary phase compared to the basal medium without MnO_2_ (Fig. 1D). In addition, the effects on MNB13 growth did not differ significantly among the groups with different MnO_2_ supplementation levels (Fig. S1). During MnO_2_ reduction by MNB13, the released Mn(II) remained at micromolar concentrations and had no significant effect on growth (Fig. S2). Collectively, these findings suggest that strain MNB13 likely diverts part of its metabolic energy to MnO_2_ reduction, resulting in decreased cellular growth.

Mn reductases, such as cellobiose dehydrogenase, are responsible for direct MnO_2_ reduction under aerobic conditions (21). However, genome analysis of strain MNB13 revealed the absence of known Mn reductases (data not shown), implicating indirect, metabolite-mediated pathways in MnO_2_ reduction (5, 21, 23). The increased proportion of Mn(III) observed during MnO_2_ reduction supports this interpretation (Fig. 1B), as indirect bioreduction of Mn(IV) typically generates a metastable Mn(III) intermediate (38–40). Furthermore, the pH of MNB13 cultures containing MnO_2_ was significantly lower than that of the culture without MnO_2_ over the incubation period (*P* < 0.05, Fig. 1E). Similar pH declines have been attributed to oxalate accumulation in *Bacillus* sp. and *Aspergillus niger* grown with MnO_2_ (41, 42). Therefore, we propose that the decreased pH in the MNB13 culture results from MnO_2_-triggered accumulation of acidic bacterial metabolites, which can also serve as electron donors for MnO_2_ reduction. Moreover, such a metabolic shift toward organic acid production would divert carbon flux away from energy production pathways, thereby supporting the observed reduction in cellular growth.

### MnO_2_ upregulates β-alanine metabolism in *Halomonas* sp. MNB13 cells

**Fig 2:**
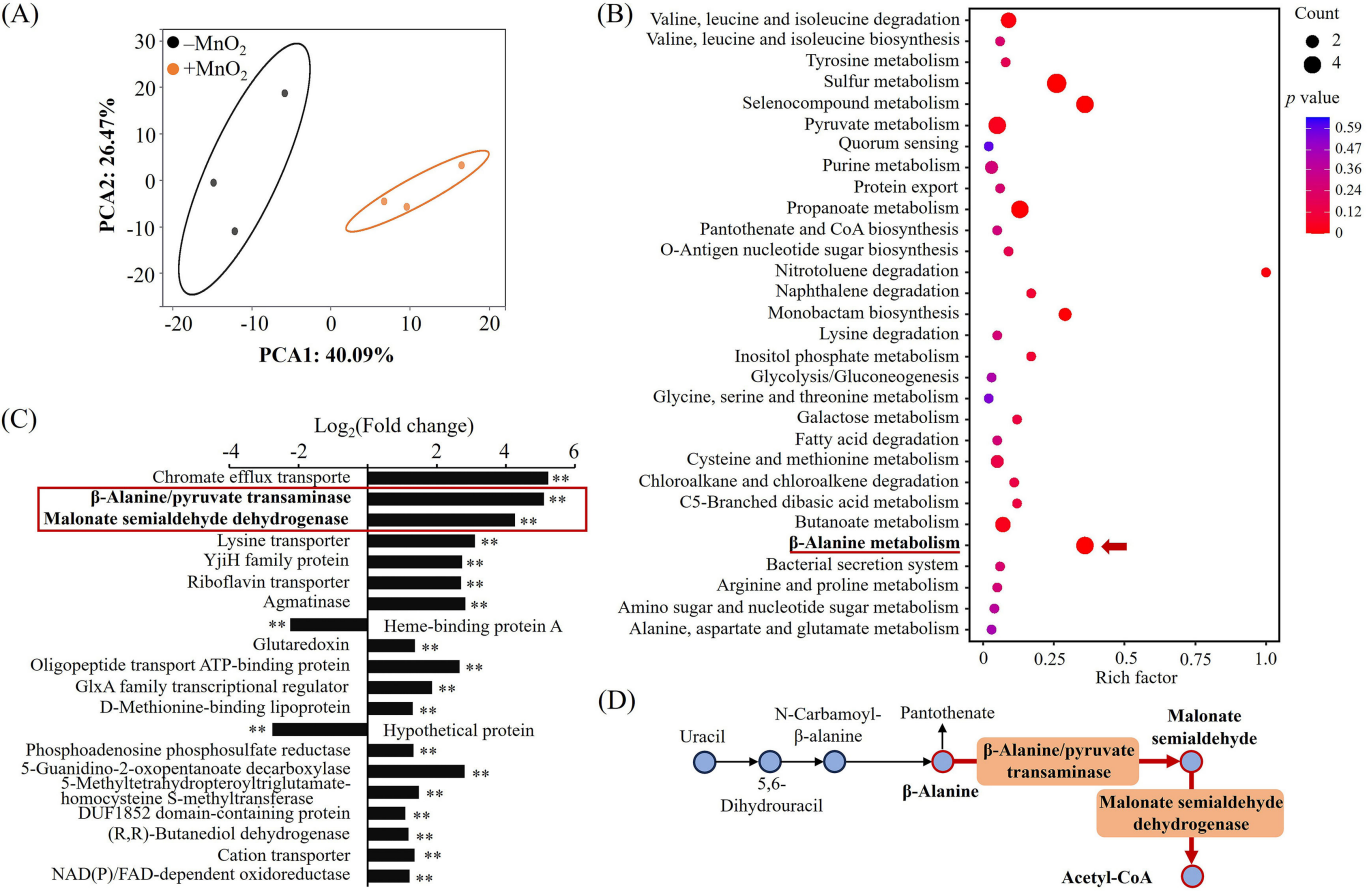
Analysis of comparative transcriptomic results *Halomonas* sp. MNB13 with (+) MnO_2_ versus without (–) MnO_2_. (**A**) Principal component analysis of detected genes in MNB13 cultures with or without MnO_2_. Triplicates of each treatment are shown. (**B**) Bubble plot of the KEGG pathway enrichment of DEGs. Bubble color and size correspond to the *P* value and gene number enriched in the pathway, respectively. The rich factor indicates the ratio of the number of DEGs mapped to a certain pathway to the total number of genes mapped to this pathway. (**C**) Fold changes of DEGs in –MnO_2_ vs +MnO_2_ (top 20 DEGs, *P* < 0.05 and |log_2_(fold change)| >1). **P < 0.01. (**D**) Schematic representation of β-alanine metabolic pathway in strain MNB13. Red, upregulated pathway. Orange, upregulated gene expression.

To elucidate the metabolic response of strain MNB13 to MnO_2_, we conducted comparative transcriptomic analyses on cells incubated with or without MnO_2_. Sequencing yielded 16.74 to 15.98 million reads for the –MnO_2_ and +MnO_2_ groups, with alignment rates >99% to the MNB13 genome (Table S1). Of the 3,635 genes annotated in the *Halomonas* sp. MNB13 genome, we detected the expression of 3,584 genes. Principal component analysis (PCA) of the global transcriptional changes showed tight clustering of biological replicates within each treatment group and clear separation between groups (total variance = 66.6%) (Fig. 2A). Pearson’s correlation and hierarchical clustering confirmed high reproducibility among replicates and distinct transcriptional profiles between treatments (Fig. S3), demonstrating that MnO_2_ induces a unique gene expression profile in MNB13 cells. We identified 80 differentially expressed genes (DEGs), including 45 downregulated and 35 upregulated genes using differential expression criteria of |log_2_(fold change)| >1 and adjusted *P* value < 0.05 (Table S2). Kyoto Encyclopedia of Genes and Genomes (KEGG) pathway analysis revealed significant enrichment in multiple metabolic pathways, particularly sulfur, β-alanine, propanoate, pyruvate, and selenocompound (Fig. 2B), each containing ≥4 DEGs. Most strikingly, two of the top three most upregulated genes encoded β-alanine/pyruvate transaminase and malonate-semialdehyde dehydrogenase (log_2_(fold change) = 5.1 and 4.3, respectively; Fig. 2C and D), strongly indicating that β-alanine metabolism is specifically upregulated in MnO_2_-exposed MNB13 cells. These observations are consistent with reports that metal oxides, such as copper and zinc oxides, can modulate microbial metabolic processes, including energy metabolism and organic acid metabolism, as well as community structure and function (43–48). In Mn-rich environments, microorganisms employ adaptive strategies, such as Mn efflux, reduction, and oxidation, to cope with metal stress (1). Our finding that MnO_2_ stimulates β-alanine metabolism in *Halomonas* sp. MNB13 reveals a previously unrecognized mechanism of Mn oxide-induced metabolic regulation in bacteria.

**Fig 3:**
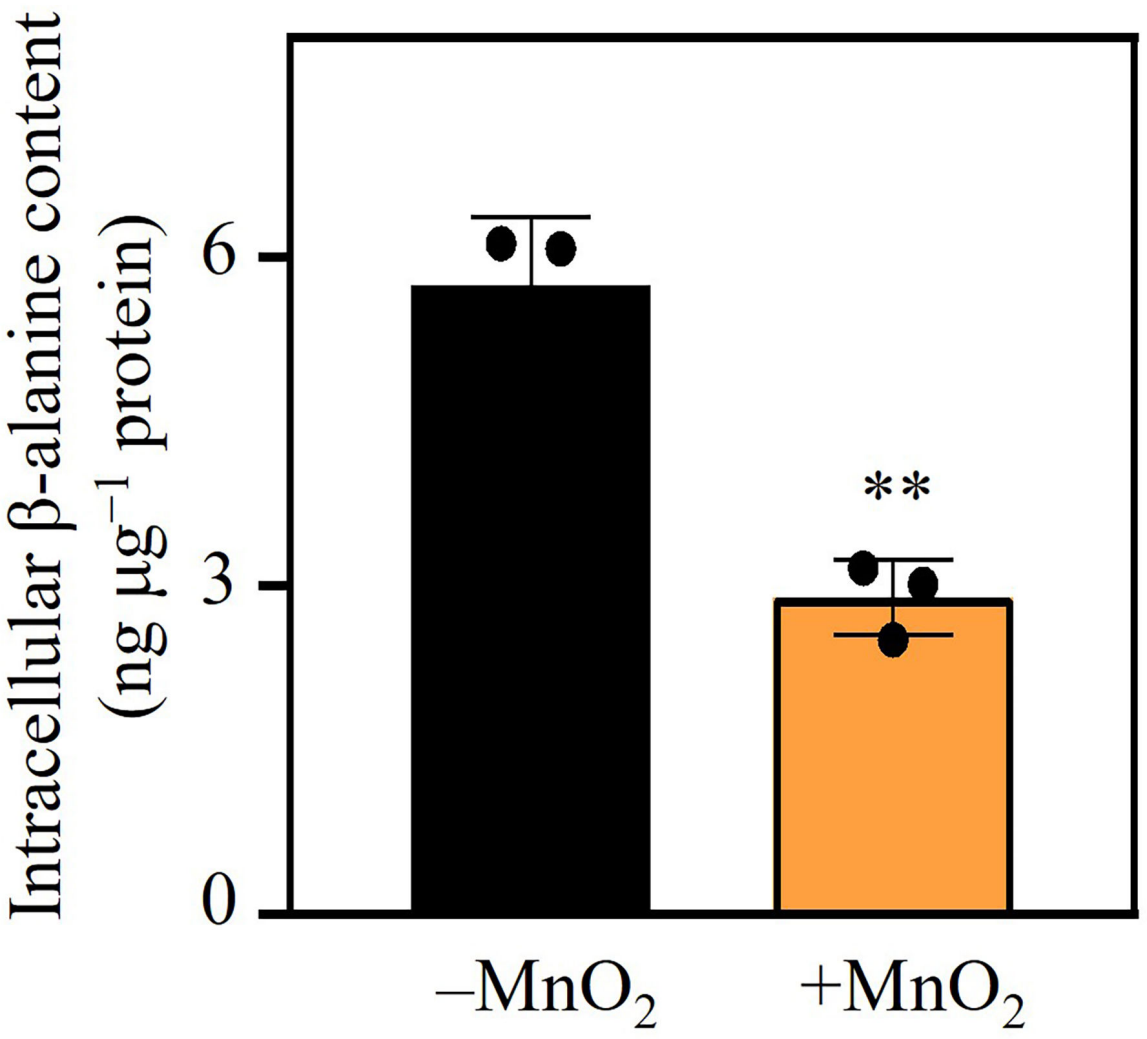
Intracellular β-alanine concentrations in *Halomonas* sp. MNB13 cells after 30 h of growth in basal medium supplemented with or without 0.5% (wt/vol) MnO_2_. **P < 0.01.

The MnO_2_-induced upregulation of β-alanine metabolism in MNB13 cells implies intracellular β-alanine depletion. Therefore, we quantified β-alanine levels in MnO_2_-exposed MNB13 cells. Consistent with the transcriptomic data, intracellular β-alanine levels decreased significantly from 5.74 ng µg^−1^ to 2.90 ng µg^−1^ protein in MnO_2_-treated cells (*P* < 0.01; Fig. 3). β-Alanine accumulation has been linked to osmoregulation in marine taxa (e.g., *Bunodosoma cavernata*, *Aurelia aurita*, and *Raja erinacea*) under high salinity via enhanced synthesis and reduced catabolism (49, 50). In contrast, the sharp decline in β-alanine level under increased Mn(II) stress argues against an osmoregulatory role in MNB13. Instead, we propose that the metabolic redirection of β-alanine may support alternative physiological functions, potentially including MnO_2_ reduction, which warrants further investigation.

### Exogenous addition of β-alanine enhances MnO_2_ reduction

**Fig 4:**
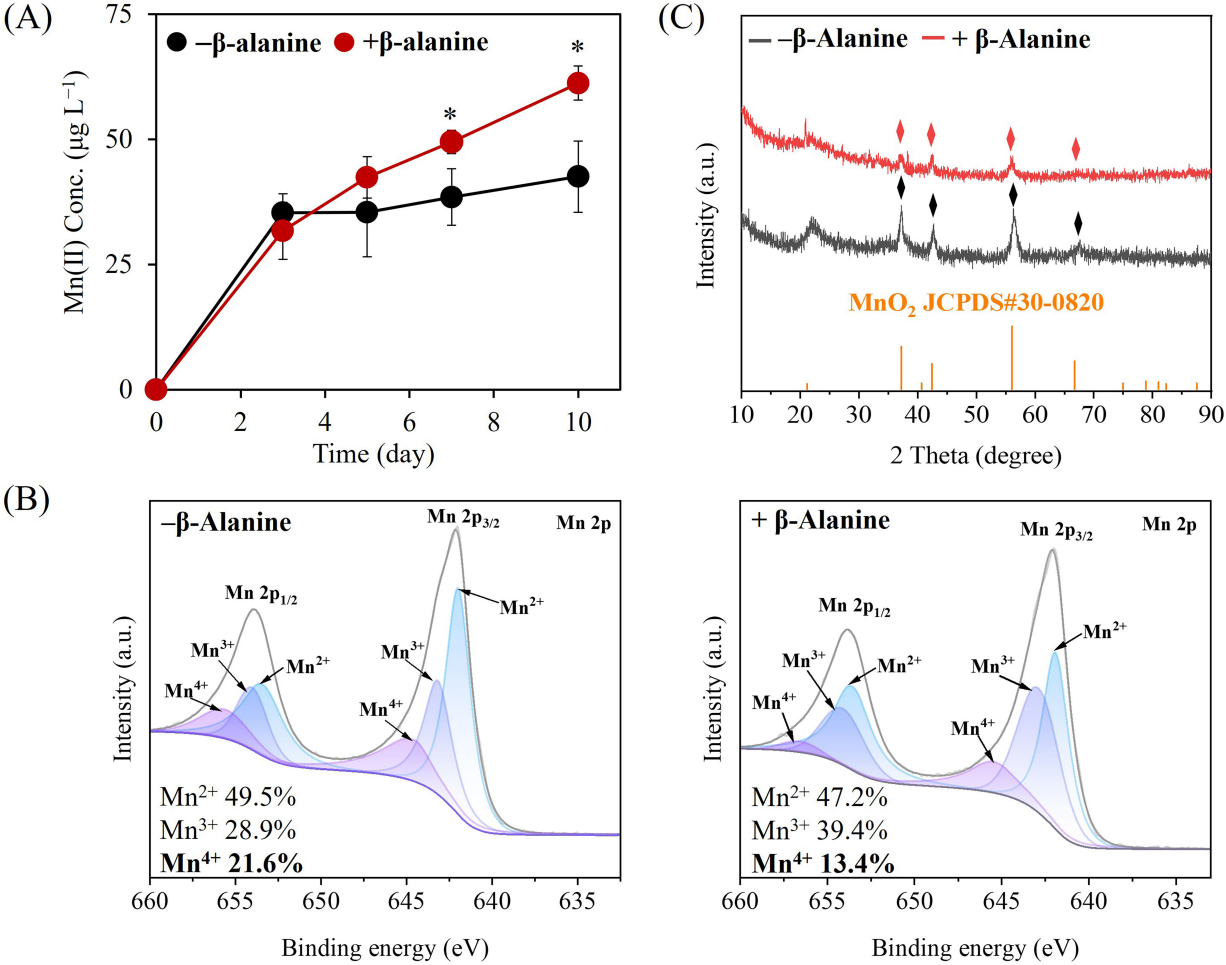
Exogenous addition of 10 mM β-alanine enhances MnO_2_ reduction in basal medium by *Halomonas* sp. MNB13. (**A**) Mn(II) concentration in the culture during MnO_2_ reduction. *P < 0.05. (**B**) Mn (2p) spectrograms of Mn residues after 28 days of cultivation. (**C**) X-ray diffraction patterns of Mn residues after 28 days of cultivation.

β-Alanine is present in deep-sea environments at concentrations ranging from 2 to 80 nmol L^−1^ (49, 51). Although bacterial contributions to Mn cycling through sulfur metabolism, carbon metabolism, and photosynthesis have been extensively documented (52–54), the relationship between environmental β-alanine and bacterial MnO_2_ reduction by bacteria has not been investigated. To examine this potential connection, we supplemented MNB13 cultures with exogenous β-alanine. Strain MNB13 encodes a TauT homolog with similarity to characterized β-alanine transporters (55). Remarkably, β-alanine supplementation enhanced MnO_2_ reduction. By day 7, Mn(II) concentrations were significantly higher in β-alanine-supplemented cultures than in controls (49.5 µg mL^−1^ vs 38.5 µg mL^−1^; *P* < 0.05; Fig. 4A), and this effect became more pronounced with prolonged incubation, reaching a maximum difference of 18.6 µg mL^−1^ by day 10 (61.2 μg mL^−1^ vs 42.6 µg mL^−1^; *P* < 0.05). These findings indicate that exogenous β-alanine significantly improves the MnO_2_ reduction capacity of strain MNB13.

To further verify β-alanine’s role in MnO_2_ reduction, we analyzed Mn residues from MNB13 cultures by XPS. The redox state distribution differed markedly between β-alanine-supplemented and control cultures (Fig. 4B). In controls, Mn residues comprised 49.5% Mn(II), 28.9% Mn(III), and 21.6% Mn(IV), whereas β-alanine-supplemented cultures exhibited 47.2% Mn(II), 39.4% Mn(III), and 13.4% Mn(IV). The notable decrease in the proportion of Mn(IV) confirms enhanced Mn(IV) reduction in the presence of β-alanine. The increase in the proportion of metastable Mn(III) intermediate supports that β-alanine indirectly enhances MNB13-mediated MnO_2_ reduction (38–40). A slight decrease in the Mn(II) proportion was also observed, potentially due to the re-adsorption of released Mn(II) onto the surface of Mn residues. Additionally, XRD analysis revealed identical characteristic peaks (ε-MnO_2_, JCPDS#30-0820) in residues from β-alanine-supplemented and unsupplemented cultures, suggesting that β-alanine did not induce the formation of new secondary Mn minerals (Fig. 4C). Taken together, exogenous β-alanine enhances MnO_2_ reduction by MNB13 through an indirect mechanism without forming new Mn mineral phases.

### β-Alanine promotes cell growth by alleviating Mn(II)-induced oxidative stress

**Fig 5:**
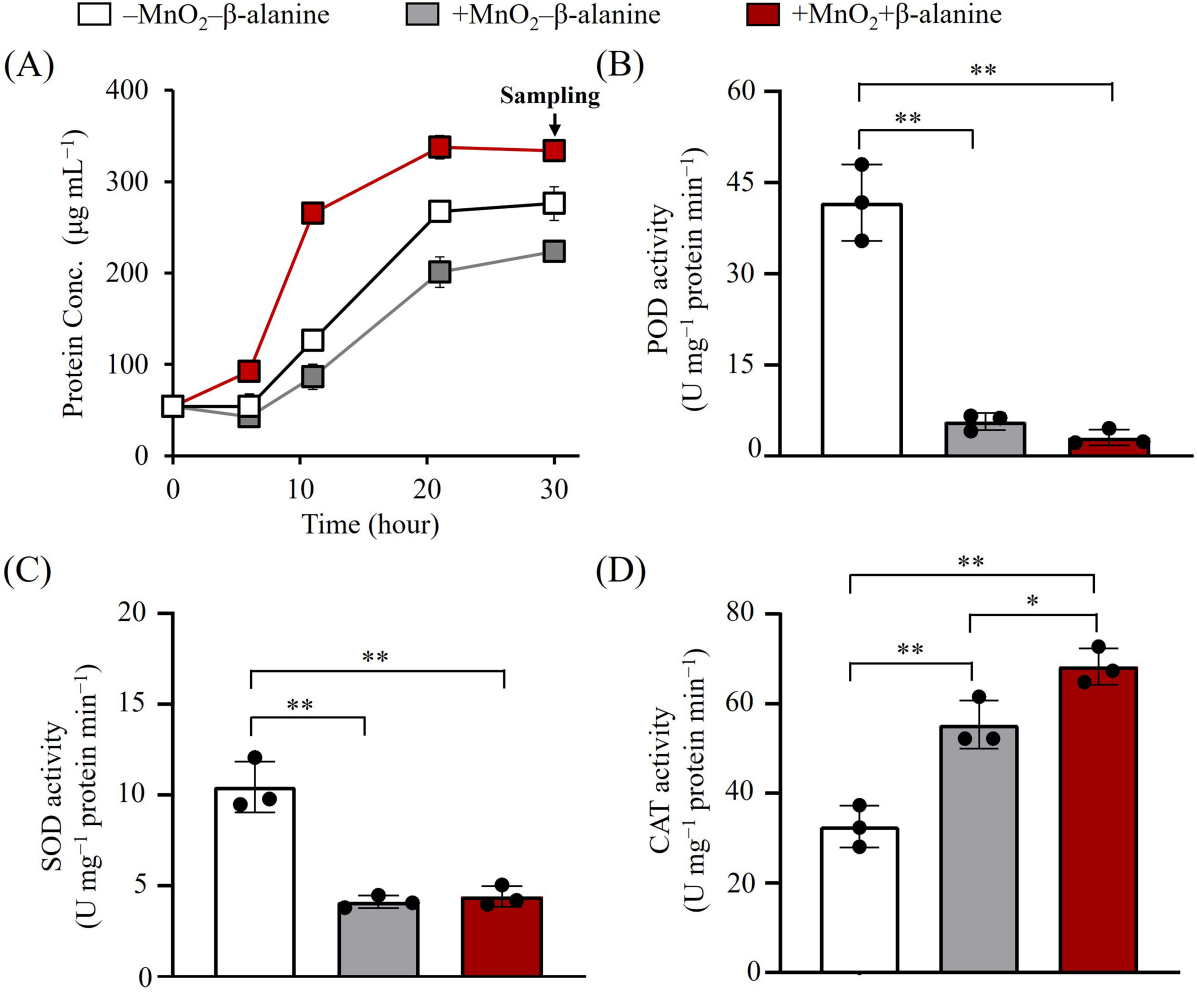
Effects of exogenous β-alanine on *Halomonas* sp. MNB13 during MnO_2_ reduction. (**A**) Growth curves of strain MNB13 under different MnO_2_ and β-alanine conditions. The arrow indicates the time points at which cultures were sampled for metabolomic analysis. (**B through D**) Intracellular antioxidant enzyme activities (POD, peroxidase; SOD, superoxide dismutase; CAT, catalase) measured after 30 h of cultivation under the specified conditions. **P < 0.01; *P < 0.05.

β-Alanine supplementation promotes the growth of aquatic animals (56–58), suggesting a potential role in supporting cellular proliferation. Given its ability to enhance MnO_2_ reduction by strain MNB13, we hypothesized that this effect might be associated with increased biomass. To test this hypothesis, we examined the influence of exogenous β-alanine on the growth of strain MNB13 during MnO_2_ reduction. β-Alanine significantly increased cell density during MnO_2_ reduction, elevating whole-cell protein concentrations from 42.9 µg mL^−1^ (+MnO_2_–β-alanine) to 92.3 µg mL^−1^ (+MnO_2_+β-alanine) at 6 h (*P* < 0.01) and from 86.4 µg mL^−1^ (+MnO_2_–β-alanine) to 265.3 µg mL^−1^ (+MnO_2_+β-alanine) at 11 h (*P* < 0.01; Fig. 5A). These results demonstrate that β-alanine supplementation restores and accelerates MNB13 growth in the presence of MnO_2_. Mechanistically, β-alanine is a utilizable carbon source (59) that can be catabolized to malonate semialdehyde via β-alanine/pyruvate transaminase and subsequently oxidized to acetyl-CoA by malonate semialdehyde dehydrogenase (Fig. 2D). Acetyl-CoA then fuels central carbon metabolism, including the Embden–Meyerhof–Parnas (EMP) pathway, pentose phosphate pathway (PPP), and tricarboxylic acid (TCA) cycle, generating ATP, reducing power, and biosynthetic precursors that support biomass accumulation.

Elevated Mn(II) levels generated during MnO_2_ reduction can induce bacterial oxidative stress (28, 60). To counteract this, bacteria upregulate antioxidant enzyme activities to maintain redox homeostasis (61). Notably, exogenous addition of β-alanine enhances catalase (CAT) and superoxide dismutase (SOD) activities in *Mytilus coruscus* cells (62). Therefore, we hypothesized that β-alanine supplementation would enhance intracellular antioxidant enzyme activity, thereby improving Mn(II) tolerance during MnO_2_ reduction and ultimately increasing cell density. To test this, we measured intracellular CAT, SOD, and peroxidase (POD) activities in MNB13 cells under different MnO_2_ and β-alanine conditions. MnO_2_ exposure significantly suppressed SOD and POD activities to 13.7% and 39.5% of control levels, respectively (*P* < 0.01; Fig. 5B and C). Strikingly, β-alanine supplementation failed to restore SOD or POD activity. In contrast, CAT activity increased 1.7-fold in the presence of MnO_2_ compared with the control (Fig. 5D). Catalase is the primary scavenger of endogenous hydrogen peroxide (63), a key mediator of heavy metal-induced oxidative stress (64). Consistent with this, we identified a gene encoding a putative Mn-dependent catalase whose activity can be enhanced by Mn(II) (65). These findings are consistent with the elevated catalase activity detected in MNB13 cells during MnO_2_ reduction, where the Mn(II) concentration progressively increased throughout the culture period. Remarkably, β-alanine further enhanced CAT activity by 2.09-fold (Fig. 5D), thereby improving Mn(II) tolerance and consequently increasing cell density. These results indicate that β-alanine differentially modulates antioxidant enzyme activities in MNB13 cells. A similar differential pattern has been observed in *M. coruscus*, where β-alanine increased SOD and CAT activities while decreasing glutathione peroxidase activity (62). Taken together, our findings indicate that β-alanine enhances MNB13 adaptation to increasing Mn(II) concentrations during MnO_2_ reduction, primarily by upregulating intracellular CAT activity. These results further suggest that β-alanine may serve as a potential redox-balancing agent to alleviate oxidative stress under MnO_2_-reducing conditions.

### β-Alanine enhances energy metabolism of MNB13 during MnO_2_ reduction

**Fig 6:**
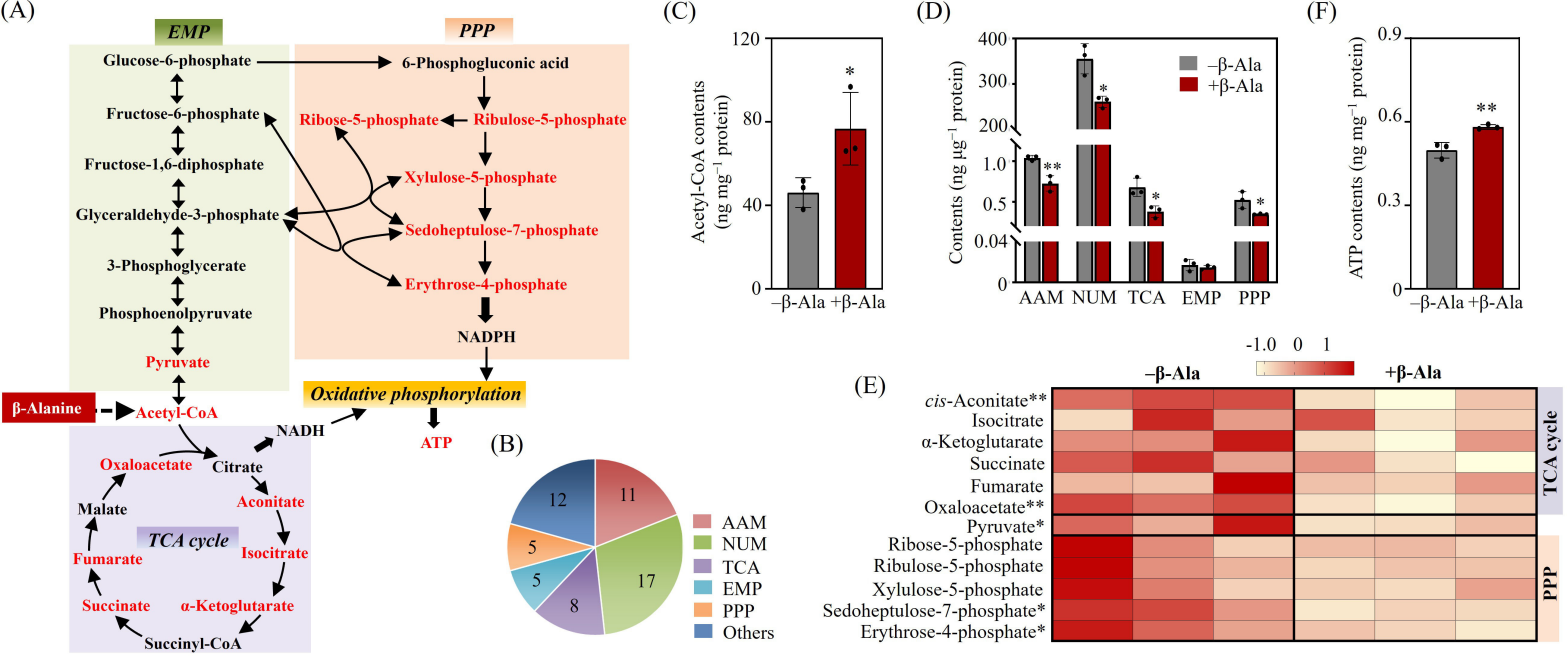
The effect of exogenous β-alanine on the metabolites of *Halomonas* sp. MNB13 during MnO_2_ reduction. (**A**) A schematic representation of energy metabolic pathways in strain MNB13. (**B**) Acetyl-CoA content. (**C**) Classification of identified metabolites gathered using UPLC-ESI-MS/MS. AAM, amino acid metabolism; NUM, nucleotide metabolism; TCA, tricarboxylic acid cycle; EMP, Embden–Meyerhof–Parnas pathway; PPP, pentose phosphate pathway. Metabolite details are listed in Table S3. (**D**) The average level of all identified metabolites in the corresponding pathway. (**E**) Heatmap showing the levels of red highlighted metabolites in energy metabolic pathways shown in Fig. 6A. (**F**) ATP content. *P < 0.05; **P < 0.01.

Exogenous β-alanine enhanced MnO_2_-reducing capacity in parallel with higher cell density, suggesting that it facilitates Mn(IV) reduction by reprogramming cellular metabolism. β-Alanine has been reported to stimulate energy metabolism in eukaryotes, including the EMP, PPP, and the TCA cycle (66–68). To systematically evaluate its effects on MNB13, we performed targeted ultra-performance liquid chromatography-electrospray ionization-tandem mass spectrometry (UPLC-ESI-MS/MS) to monitor flux changes across central carbon metabolism (Fig. 6A). The PCA analysis revealed clear separation between β-alanine-treated and untreated cells, accounting for 65.7% of total variance, indicating distinct metabolic profiles (Fig. S4). We identified 58 metabolites associated with the EMP, PPP, TCA cycle, amino acid metabolism (AAM), and nucleotide metabolism (NUM) (Fig. 6B; Table S3).

β-Alanine metabolism directly increased intracellular acetyl-CoA content via β-alanine/pyruvate transaminase and malonate-semialdehyde dehydrogenase (Fig. 2D). This was quantitatively confirmed by a significant increase in acetyl-CoA content from 46.1 ng to 76.7 ng per μg protein after the addition of β-alanine (*P* < 0.05; Fig. 6C), suggesting that strain MNB13 can efficiently transport and metabolize β-alanine. Under MnO_2_ conditions, the addition of β-alanine significantly decreased the amounts of multiple TCA cycle and PPP intermediates (Fig. 6D), notably *cis*-aconitate and oxaloacetate (TCA cycle) and sedoheptulose-7-phosphate and erythrose-4-phosphate (PPP) (Fig. 6E). Additionally, β-alanine-treated cells also exhibited significantly reduced pyruvate levels in the EMP (Fig. 6E). This coordinated depletion pattern implies accelerated metabolic flux through these pathways, likely driven by the heightened demand for reducing equivalents and ATP. In line with this, intracellular ATP levels increased from 0.50 ng mg^−1^ protein to 0.58 ng mg^−1^ protein (*P* < 0.01) (Fig. 6F). In summary, these results indicate that β-alanine enhances MNB13 cell growth during MnO_2_ reduction by stimulating energy metabolism pathways, promoting acetyl-CoA production, and increasing ATP levels.

### Pantothenate and levulinate play roles in MnO_2_ reduction

**Fig 7:**
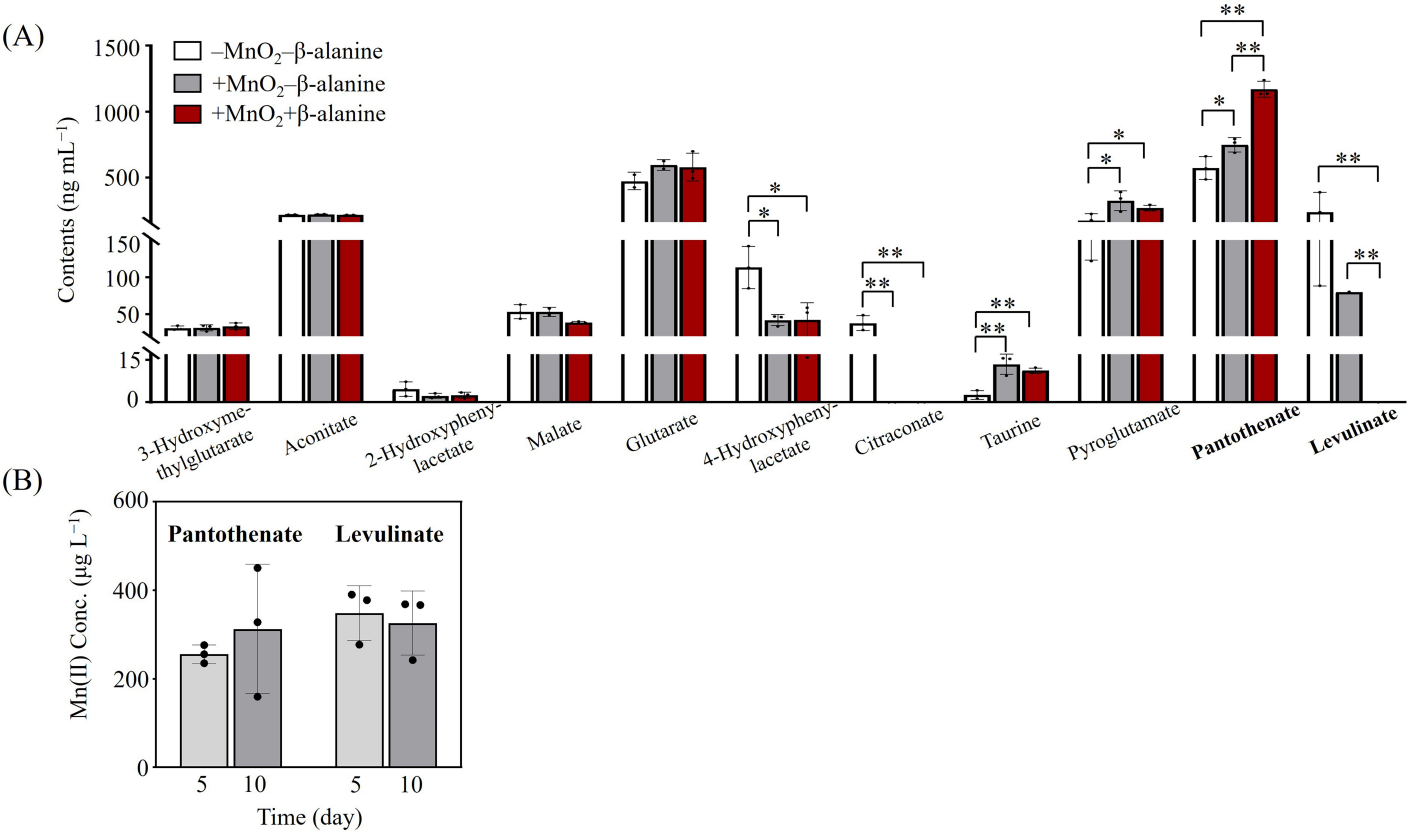
Identified organic acids associated with MnO_2_ reduction by *Halomonas* sp. MNB13. (**A**) Concentrations of organic acids detected in the culture supernatants of MNB13. *P < 0.05; **P < 0.01. (**B**) Mn(II) release from MnO_2_ after incubation MnO_2_ with pantothenate (1,000 ng mL^−1^) and levulinate (300 ng mL^−1^).

Organic acids secreted by bacteria can facilitate Mn oxide reduction (5, 21, 23). Consistent with the decreased cultural pH in MnO_2_-containing cultures (Fig. 1C), we identified 11 organic acids in the culture supernatant whose concentrations varied by orders of magnitude (Fig. 7A). Notably, commonly reported organic acids associated with MnO_2_ reduction (e.g., oxalate, citrate, fumarate, acetate, pyruvate, and lactate) were not detected in MNB13 cultures (7, 69–71). This suggests that strain MNB13 utilizes alternative metabolites for medium acidification and MnO_2_ reduction. The concentrations of 3-hydroxymethylglutarate, aconitate, 2-hydroxyphenylacetate, malate, and glutarate did not differ significantly among the three treatments and are thus unlikely to contribute directly to β-alanine-associated MnO_2_ reduction (*P* > 0.05). Additionally, 4-hydroxyphenylacetate and citraconate significantly decreased in the presence of MnO_2_ compared with their levels in the absence of MnO_2_, and β-alanine supplementation did not restore their concentrations. Conversely, the addition of MnO_2_ increased the levels of taurine and pyroglutamate, which remained unchanged upon β-alanine supplementation. These findings suggest that the β-alanine enhanced MnO_2_ reduction by strain MNB13 is not mediated, or only minimally influenced, by these acids.

Strikingly, the pantothenate level increased in the presence of MnO_2_ (*P* < 0.05) and was further elevated with β-alanine supplementation (*P* < 0.01). Specifically, pantothenate content was 1.56-fold higher after β-alanine addition compared with cultures without β-alanine in the presence of MnO_2_. Although pantothenate is known for its roles in energy metabolism, gene expression regulation, cell wall synthesis, and antioxidant protection (24), its involvement in Mn reduction has not been reported. Given that pantothenate can be synthesized from β-alanine by pantoate-β-alanine ligase in MNB13, exogenous β-alanine likely drives the observed pantothenate accumulation in the culture system. To test whether pantothenate mediates MnO_2_ reduction, we co-incubated 1,000 ng mL^−1^ of pantothenate with MnO_2_ in a basal medium. After five days, pantothenate enhanced the release of Mn(II) from MnO_2_ (Fig. 7B), indicating its role as an electron donor for MnO_2_ reduction. However, the Mn(II) concentration was not statistically different on day 10 compared with that on day 5, likely due to the re-adsorption of dissolved Mn(II) onto the residual Mn oxides. Additionally, a significant decrease in levulinate levels was observed in the presence of MnO_2_, which further declined after β-alanine supplementation. A feeding experiment confirmed that levulinate increased Mn(II) release during co-incubation with MnO_2_ (Fig. 7B), implicating levulinate as an additional electron donor, consistent with its reduced concentration in solution (Fig. 7A). In summary, these results indicate that pantothenate and levulinate contribute to the enhanced MnO_2_ reduction observed with β-alanine addition, with pantothenate likely playing a particularly significant role in the associated pH decrease. However, a comprehensive study into the detailed reduction mechanism was not conducted. Further studies are warranted to determine how pantothenate and levulinate supply electrons to reduce Mn(III/IV).

### Conclusions

**Fig 8:**
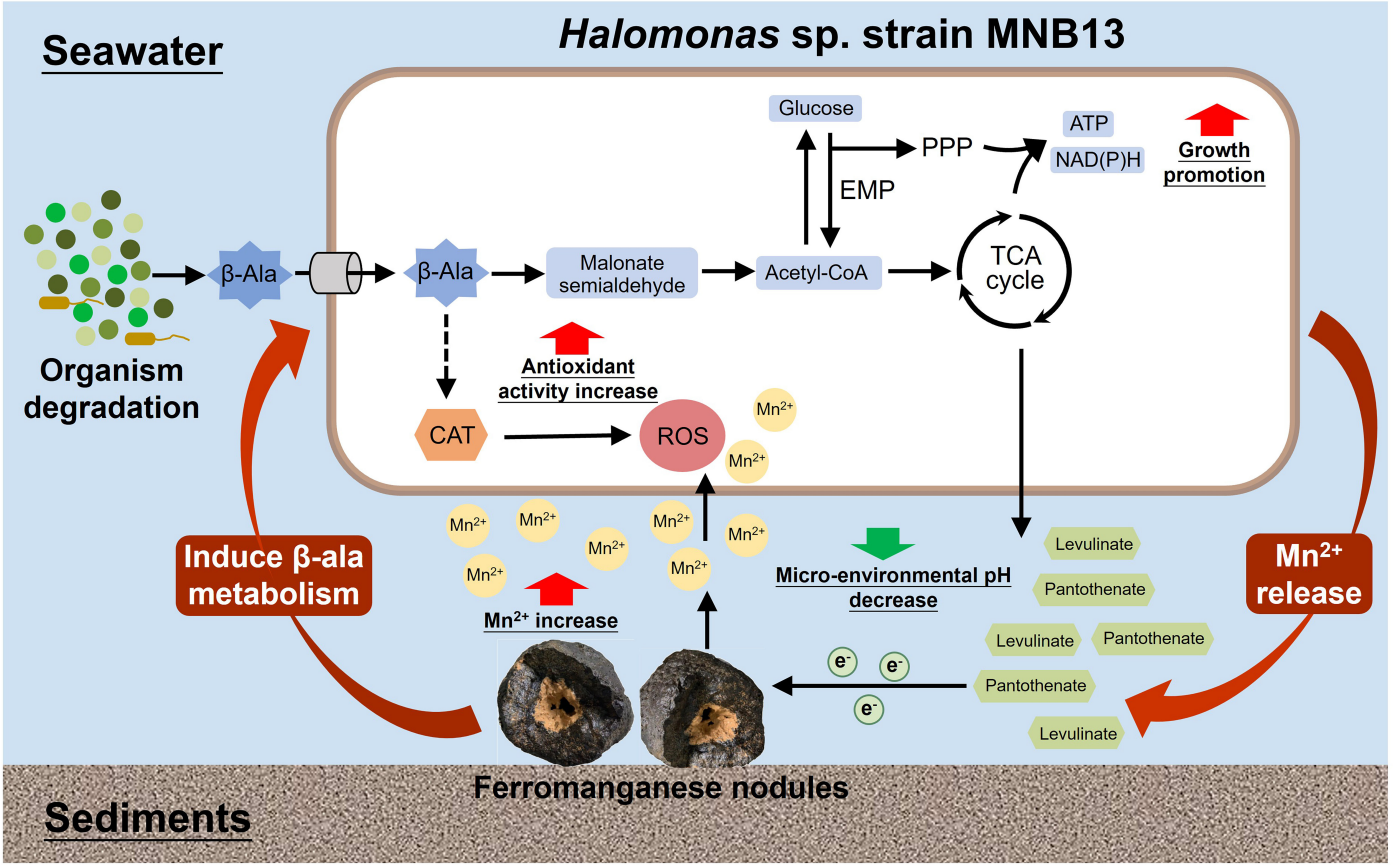
Schematic illustration depicting strain MNB13’s utilization of β-alanine to facilitate Mn(II) release from ferromanganese nodules in the deep-sea environment.

*Halomonas* sp. MNB13, isolated from a deep-sea ferromanganese nodule, releases Mn(II) from MnO_2_ through an indirect, metabolism-mediated mechanism (Fig. 8). Transcriptomic analysis revealed that exposure to MnO_2_ significantly upregulated β-alanine metabolism, corroborated by reduced intracellular β-alanine levels. Exogenous β-alanine promoted MNB13 growth and reduced the proportion of Mn(IV) without altering the type of Mn residues. Enhanced growth was achieved by increasing energy metabolism, including stimulation of the EMP pathway, PPP, and TCA cycle, leading to increased ATP production. Simultaneously, intracellular catalase activity was also upregulated, consistent with the need to eliminate reactive oxygen species generated upon Mn(II) release. Furthermore, β-alanine addition reshaped the profile of secreted organic acids, and we confirmed that two of them, pantothenate and levulinate, were responsible for the enhanced Mn(IV) reduction. These findings describe how bacterial metabolism of β-alanine facilitates Mn(II) release from deep-sea ferromanganese nodules, highlighting the ecological importance of non-proteinogenic amino acids in the deep-sea Mn cycle.

## MATERIALS AND METHODS

### Bacterial strain and cultivation

Strain MNB13 was isolated from a deep-sea ferromanganese nodule sample collected by the Kexue vessel (NORC2020-581, 2020–2021) at the coordinates 139°33′24.638′′E, 14°03′15.051″N and was identified as *Halomonas* sp. MNB13 (28). Strain MNB13 was cultured aerobically in Luria-Bertani medium at 28°C with continuous shaking at 150 rpm. Cells in the exponential phase were harvested for MnO_2_ reduction experiments. For the MnO_2_ reduction assays, strain MNB13 was grown in a basal medium (per liter of distilled water) consisting of 1.67 g NH_4_Cl, 3.16 g KNO_3_, 1.5 g KH_2_PO_4_, 0.1 g CaCl_2_, 1.02 g MgSO_4_·7H_2_O, and 0.5 g yeast extract. The medium was adjusted to a pH of 7.0 prior to sterilization. The composition was modified from a previously described medium by Toro et al. (72). The growth of strain MNB13 was quantified by measuring the total protein concentration per milliliter. Briefly, 2 mL of bacterial cells were harvested by centrifugation (8,000 × *g* for 10 min at 4°C) and resuspended in phosphate buffer (pH 7.0–7.2). Cells were lysed by sonication (3 s pulse, 5 s rest intervals, 50 cycles; Xinzhi, China) on ice and centrifuged at 12,000 × *g* for 10 min at 4°C. The protein content in the supernatant was then determined using a bicinchoninic acid assay kit (Cat. No. A045-4, Nanjing Jiancheng Bioengineering Institute, Nanjing, China) according to the manufacturer’s instructions.

### MnO_2_ reduction assay

The MnO_2_ reduction assays were performed in the basal medium. A commercial ε-MnO_2_-like powder of mixed valence (JCPDS#30-0820), containing Mn(II), Mn(III), and Mn(IV), was used as the sole added MnO_2_ sources. Strain MNB13 was inoculated into the 50 mL basal medium with 0.5% MnO_2_ (wt/vol) in a 150 mL flask and incubated aerobically at 28°C with shaking at 150 rpm. Dissolved Mn(II) concentrations were determined at specified time points using inductively coupled plasma mass spectrometry (iCAP Q, Thermo Fisher Scientific, USA).

### Comparative transcriptomic analysis

Mid-log-phase cells of strain MNB13 cultured in LB medium were collected by centrifugation (8,000 × *g* for 10 min at 4°C), washed with basal medium three times, and then resuspended in basal medium, either with (+) or without (–) 0.5% MnO_2_. After 30 h of incubation at 28℃ with continuous shaking at 150 rpm, the cells were harvested and washed thrice with ice-cold 0.85% NaCl. Total RNA was extracted using TRIzol reagent according to the manufacturer’s instructions. RNA quality was evaluated using a Thermo NanoDrop One and Agilent 4200 Tape Station. Ribosomal RNA was removed using the Ribo-Zero rRNA Removal Kit (Epicenter, Illumina Inc.), and cDNA libraries were constructed using the NEB Next Ultra II Directional RNA Library Prep kit (Illumina, USA). Sequencing was performed in rapid mode with a 150 nt read length (Illumina PE150, USA; Guangdong Magigene Biotechnology Co., Ltd., China). Clean reads were obtained by removing sequences with overrepresented adaptors, short reads (<75 nt), pair-end reads with >5% “N” bases, and reads with over 20% bases having a quality score less than 20. All clean reads were aligned to the genomic sequence of MNB13 using Bowtie2. Transcriptomic analysis was performed as described by Song et al. (73). DEGs (|log_2_(fold change)| >1 and adjusted *P* value < 0.05) in the comparison of –MnO_2_ vs +MnO_2_ were enriched in KEGG pathways (https://www.kegg.jp/).

### Measurement of intracellular β-alanine

The samples collected for transcriptome analysis were also used to measure intracellular β-alanine levels. Bacterial cells were resuspended in phosphate buffer (pH = 7.0–7.2), lysed by sonication (3 s pulse, 5 s rest intervals, 50 cycles) on ice, and centrifuged (12,000 × *g* for 10 min at 4°C). The supernatant (800 µL) was mixed with 400 µL each of derivative agents A (triethylamine:acetonitrile = 139:861) and B (phenylisothiocyanate:acetonitrile = 12:988) and incubated at 26°C for 1 h. Following this, 1.6 mL of butanol was added, and the mixture was re-centrifuged as described above. The sample was then analyzed using high-performance liquid chromatography equipped with a C18 column (Phenomenex Aqua, 4.6 × 250 mm, 5 µm). Separation was achieved with a two-phase mobile system at a flow rate of 1 mL min^−1^, with solvent A consisting of 97:3 acetonitrile:0.1 M sodium acetate solution (pH 7.0), and solvent B was 4:1 being acetonitrile:water. The gradient elution program was as follows: 0–30 min, 95–70% A; 30–35 min, 70–95% A. The column temperature was maintained at 40°C, and the injection volume was 20 µL. Chromatograms were recorded at 254 nm, and β-alanine content was quantitatively determined by comparing the chromatographic peak areas with a standard curve of known β-alanine concentrations.

### Examination of Mn residues

Strain MNB13 was aerobically cultivated in basal medium supplemented with or without 10 mM β-alanine in the presence of 0.5% MnO_2_ at 28°C for 28 days. The Mn residues were collected by centrifugation (8,000 × *g* for 10 min at 26°C) and freeze-dried at −96°C. XRD analysis was performed by scanning the Mn residues with a 2*θ* range of 10-90°, using an X-ray diffractometer (KYOWAGLAS-XA H-12, Rigaku, Japan). The valence state of Mn was determined by XPS (ESCALAB Xi+, Thermo Fisher Scientific, USA).

### Antioxidant enzyme activity assay

*Halomonas* sp. MNB13 cells were incubated with or without 10 mM β-alanine in the presence of 0.5% MnO_2_ at 28℃ for 30 h to assess the activities of intracellular CAT, SOD, and POD. Then, the cells were collected and resuspended in phosphate buffer (pH = 7.0–7.2), lysed by sonication (3 s pulse, 5 s rest intervals, 50 cycles) on ice, and centrifuged at 12,000 × *g* for 10 min at 4°C. The supernatants were assayed for antioxidant enzyme activity assay using specific assay kits (CAT, BC0200; SOD, BC0170; POD, Solarbio, Beijing, China) according to the manufacturer’s instructions.

### Detection of intracellular energy metabolism-related metabolites

Mid-log-phase cells of strain MNB13 were collected by centrifugation (5,000 × *g* for 10 min at 26°C) and resuspended in a basal medium containing 0.5% MnO_2_, either with or without 10 mM β-alanine. The samples were designated as –β-alanine (without β-alanine) and +β-alanine (with β-alanine), respectively. After 30 h of incubation at 28℃ with continuous shaking at 150 rpm, the cells were harvested (8,000 × *g* for 10 min at 4℃), washed thrice with ice-cold 0.85% NaCl, and then freeze-dried at −80℃. The resulting samples were ground and dissolved in 500 µL of 70% methanol. Metabolites were extracted by shaking at 1,500 rpm for 5 min at 4°C, followed by centrifugation (12,000 × *g* for 10 min at 4℃). A 300 µL aliquot of the supernatant underwent protein precipitation by incubation at −20℃ for 30 min (74). After re-centrifugation, the supernatant was analyzed using an UPLC-ESI-MS/MS system (UPLC, ExionLC AD; MS, QTRAP 6500+, Sciex) equipped with an Acquity UPLC BEH amide column (2.1 × 100 mm, 1.7 µm). The analytical procedure employed a two-phase mobile system at a flow rate of 0.4 mL min^−1^, with solvent A consisting of 10 mM of ammonium acetate and 0.3% ammonia, and solvent B consisting of 9:1 acetonitrile:water. The gradient elution program was as follows: 0–1.2 min, 5% A; 1.3–8 min, 30% A; 9–11 min, 50% A; and 11.1–15 min, 5% A. Metabolite analysis (qualitative and quantitative) was conducted using self-built MWDB databases (Metware Biotechnology Co., Ltd., China).

### Detection of secreted organic acids in culture

Mid-log-phase cells of strain MNB13 cultured in LB medium were collected by centrifugation at 8,000 × *g* for 10 min at 26°C, washed with basal medium three times, and then resuspended in basal medium with or without β-alanine in the presence of MnO_2_. After 30 hours of incubation at 28℃ with continuous shaking at 150 rpm, cultures were centrifuged (8,000 × *g* for 10 min at 4°C) to obtain supernatants for organic acid analysis. Each sample (50 µL) was mixed with 250 µL of extract solution (20:80 acetonitrile:methanol), and agitated at 1,500 rpm for 5 min at 4℃ to extract the metabolites. After centrifugation (12,000 × *g* for 10 min at 4℃), 250 µL of the supernatant was incubated at −20℃ for 30 min to precipitate proteins (70). The supernatant was then re-centrifuged as described above and analyzed using the UPLC-ESI-MS/MS system with an Acquity HSS T3 column (2.1 × 100 mm, 1.8 µm). The analytical conditions included a two-phase mobile system at a flow rate of 0.35 mL min^−1^, with solvent A being water with 0.05% formic acid and solvent B being acetonitrile with 0.05% formic acid. The gradient elution program was as follows: 0–8 min, 5–95% B; 8–9.5 min 95% B; and 9.5–12 min, 5% B. The column temperature was maintained at 40°C, and the injection volume was 2 µL. Metabolites were qualitatively and quantitatively analyzed using the self-built MWDB databases (Metware Biotechnology Co., Ltd.).

### Statistical analyses

All experiments were performed in triplicate. PCA was performed using the statistical function prcomp in R (https://www.r-project.org/). Data are presented as the mean ± standard deviation. One-way analysis of variance was used to identify differences between experimental groups, with significance levels indicated as follows: **P < 0.01; *P < 0.05.

## Data Availability

The raw sequencing data from this study have been deposited in the Genome Sequence Archive in BIG Data Center, Beijing Institute of Genomics (BIG), Chinese Academy of Sciences, under the accession number: CRA033181 (https://ngdc.cncb.ac.cn/gsa/browse/CRA033181. A, –MnO_2_; B, +MnO_2_).

## SUPPLEMENTAL MATERIAL

Supplemental material aem.02164-25-s0001.docx Tables S1 and S2; Fig. S1 to S4.

ASM does not own the copyrights to Supplemental Material that may be linked to, or accessed through, an article. The authors have granted ASM a non-exclusive, world-wide license to publish the Supplemental Material files. Please contact the corresponding author directly for reuse.
