# Supplementary material for: Non-proteinogenic β-alanine metabolism in Halomonas sp. MNB13 regulates manganese reduction in deep-sea ferromanganese nodules
Source: Appl Environ Microbiol. 2025 Nov 18;91(12):e02164-25. doi: 10.1128/aem.02164-25 (PMC12724341; doi:10.1128/aem.02164-25)
Supplement: Supplemental material — Tables S1 and S2; Fig. S1 to S4. [file aem.02164-25-s0001.docx]

**Non-proteinogenic β-alanine metabolism in *Halomonas* sp. MNB13 regulates manganese reduction** **in deep-sea ferromanganese nodules**

Running title: β-alanine triggers Mn(Ⅳ) reduction of ferromanganese

Shuju Guo^1,2#^, Xiuli Xu^3#^, Hui Shuai^4,5^, Fuhang Song^6^, Guoliang Zhang^7^, Linlin Ma^8^, Na Yang^1,2*^

^1^ Laboratory of Experimental Marine Biology, Institute of Oceanology, Chinese Academy of Sciences, 88 Haijun Road, Qingdao, 266400, P. R. China

^2^ Laboratory for Marine Biology and Biotechnology, Qingdao National Laboratory for Marine Science and Technology, 1 Wenhai Road, Qingdao, 266237, P. R. China

^3^ Key Laboratory of Marine Mineral Resources and Polar Geology, Ministry of Education of China; School of Ocean Sciences, China University of Geosciences, 29 Xueyuan Road, Beijing, 100083, P. R. China

^4^ Department of Marine Technology, Rizhao Polytechnic, Rizhao, 276826, P. R. China

^5^ State Key Laboratory of Integration and Innovation of Classic Formula and Modern Chinese Medicine, Lunan Pharmaceutical Group Co. Ltd., Linyi, 276005, P. R. China

^6^ School of Light Industry, Beijing Technology and Business University, 11 Fucheng Road, Beijing, 100048, P. R. China

^7^ School of Marine Sciences, Sun Yat-sen University, Zhuhai, P. R. China

^8^ Institute for Biomedicine and Glycomics, School of Environment and Science, Griffith University, Brisbane, 4111, Australia

^*^Corresponding author:

Na Yang. Laboratory of Experimental Marine Biology, Institute of Oceanology, Chinese Academy of Sciences, 88 Haijun Road, Qingdao, 266400, P. R. China. E-mail address: yangna@qdio.ac.cn (N. Yang). Telephone: 86-532-82876007

**^#^**Shuju Guo and Xiuli Xu contributed equally to this article.

**Table S1.** Summary of RNA sequencing and mapped reads.

| Sample | Total reads | Paired mapped reads | Total mapped reads | Total mapped ratio (%) |
| --- | --- | --- | --- | --- |
| –MnO_2_-1 | 17000290 | 16940228 | 16967883 | 99.81 |
| –MnO_2_-2 | 18214772 | 18117732 | 18149101 | 99.64 |
| –MnO_2_-3 | 14990638 | 14939710 | 14962078 | 99.81 |
| +MnO_2_-1 | 17406196 | 17338288 | 17367690 | 99.78 |
| +MnO_2_-2 | 18235364 | 18162902 | 18196095 | 99.78 |
| +MnO_2_-3 | 12289938 | 12130824 | 12198891 | 99.26 |

**Table S2.** Differentially expressed genes in comparisons of –MnO_2_ *vs.* +MnO_2_.

| Gene | log_2_(fold change) | *p* value | Name | Description |
| --- | --- | --- | --- | --- |
| GE002241 | 5 227105 | 6 47E-181 | WP_113271274 1 | Chromate efflux transporter |
| GE003131 | 5 100575 | 2 63E-65 | WP_153843006 1 | Aspartate aminotransferase family protein |
| GE003130 | 4 262935 | 6 53E-59 | NQY76690 1 | Coa-acylating methylmalonate-semialdehyde dehydrogenase |
| GE002912 | 3 10448 | 3 55E-32 | MAO61446 1 | Lysine transporter lyse |
| GE003035 | 2 730242 | 9 67E-27 | WP_139525761 1 | Yjih family protein |
| GE003065 | 2 702299 | 5 36E-23 | WP_153843043 1 | MFS transporter |
| GE003036 | 2 824805 | 1 02E-20 | WP_139525762 1 | Agmatinase |
| GE000574 | -2 23871 | 1 73E-20 | WP_139525079 1 | ABC transporter substrate-binding protein |
| GE003434 | 1 365855 | 3 40E-14 | WP_219862217 1 | Glutaredoxin |
| GE000491 | 2 65869 | 2 32E-13 | WP_139528366 1 | ABC transporter ATP-binding protein |
| GE003066 | 1 86744 | 5 01E-13 | WP_239297063 1 | Glxa family transcriptional regulator |
| GE001521 | 1 309458 | 2 90E-11 | WP_139528058 1 | Metq/nlpa family ABC transporter substrate-binding protein |
| GE002815 | -2 7541 | 3 02E-11 | CAD5253492 1 | Hypothetical protein HALO59_120437 |
| GE001214 | 1 322618 | 8 31E-11 | WP_139525596 1 | Phosphoadenosine phosphosulfate reductase family protein |
| GE003034 | 2 804449 | 1 37E-10 | WP_218013965 1 | 5-Guanidino-2-oxopentanoate decarboxylase |
| GE002884 | 1 480241 | 4 73E-10 | WP_153842488 1 | 5-Methyltetrahydropteroyltriglutamate--homocysteine S-methyltransferase |
| GE002568 | 1 094172 | 6 10E-10 | WP_153842665 1 | DUF1852 domain-containing protein |
| GE001414 | 1 181556 | 1 35E-09 | BCB61931 1 | (R,R)-butanediol dehydrogenase |
| GE002242 | 1 357417 | 1 99E-09 | WP_074211754 1 | CDF family Co(Ⅱ)/Ni(Ⅱ) efflux transporter dmef |
| GE001413 | 1 213339 | 2 51E-09 | WP_239297227 1 | NAD(P)/FAD-dependent oxidoreductase |
| GE000062 | -1 18415 | 6 95E-09 | WP_139528561 1 | TRAP transporter fused permease subunit |
| GE002229 | 1 150777 | 8 63E-09 | WP_219861168 1 | Sulfate adenylyltransferase subunit cysn |
| GE001689 | 1 187955 | 1 29E-08 | WP_228168447 1 | Nitrite/sulfite reductase |
| GE002911 | 1 084532 | 1 65E-08 | WP_153842482 1 | PLP-dependent aminotransferase family protein |
| GE000217 | 1 450641 | 5 64E-08 | WP_239296414 1 | ABC transporter ATP-binding protein |
| GE002240 | 1 177391 | 9 22E-08 | WP_113271275 1 | Chromate resistance protein |
| GE000710 | -2 5307 | 1 55E-07 | WP_219862991 1 | SO_0444 family Cu/Zn efflux transporter |
| GE001688 | 1 174394 | 1 60E-07 | WP_153843327 1 | DUF934 domain-containing protein |
| GE003433 | 1 6396 | 2 99E-07 | KFC50807 1 | Hypothetical protein DK37_06570 |
| GE001196 | 1 167742 | 6 84E-07 | WP_153842416 1 | Thiosulfate ABC transporter substrate-binding protein cysp |
| GE000024 | -1 45678 | 7 67E-07 | WP_239286352 1 | Bifunctional glycoside hydrolase 114/ polysaccharide deacetylase family protein |
| GE001955 | -2 166 | 7 73E-07 | WP_089037843 1 | DUF4942 domain-containing protein |
| GE000801 | -1 51701 | 9 93E-07 | WP_153842223 1 | CZB domain-containing protein |
| GE000025 | -1 72582 | 1 07E-06 | WP_139526193 1 | NAD-dependent epimerase/dehydratase family protein |
| GE003033 | 1 63649 | 1 21E-06 | WP_139525760 1 | Aldehyde dehydrogenase family protein |
| GE002230 | 1 091508 | 1 49E-06 | WP_153842810 1 | Sulfate adenylyltransferase subunit cysd |
| GE003669 | -1 68083 | 1 48E-06 | WP_139526161 1 | Csgg/hfab family protein |
| GE000841 | 1 205482 | 1 74E-06 | WP_153842251 1 | C4-dicarboxylate ABC transporter substrate-binding protein |
| GE000073 | -1 50497 | 2 10E-06 | WP_153843278 1 | SDR family NAD(P)-dependent oxidoreductase |
| GE000143 | -1 29878 | 2 07E-06 | WP_153843254 1 | Cyclopropane-fatty-acyl-phospholipid synthase family protein |
| GE002000 | -2 32468 | 2 44E-06 | HBP79860 1 | Hypothetical protein |
| GE002002 | -1 85942 | 2 87E-06 | WP_206047529 1 | DUF2184 domain-containing protein |
| GE002008 | -2 27373 | 3 28E-06 | WP_162527873 1 | Phage tail tube protein |
| GE003071 | -1 12864 | 3 28E-06 | WP_139525789 1 | Malonyl-coa decarboxylase |
| GE002324 | 1 384553 | 3 62E-06 | WP_239286433 1 | Porin |
| GE000215 | 1 220928 | 3 76E-06 | WP_219862402 1 | Zinc ABC transporter substrate-binding protein |
| GE000800 | -1 06506 | 3 86E-06 | WP_153842222 1 | Patatin-like phospholipase family protein |
| GE001963 | -1 87576 | 4 40E-06 | PHS59666 1 | Phage recombination protein Bet |
| GE001311 | -1 30917 | 5 40E-06 | WP_139528450 1 | Outer membrane beta-barrel protein |
| GE000575 | -1 02564 | 5 58E-06 | WP_062373147 1 | Gntr family transcriptional regulator |
| GE002227 | 1 052352 | 6 91E-06 | WP_153842813 1 | ABC transporter substrate-binding protein |
| GE002270 | -1 3736 | 7 48E-06 | WP_139527050 1 | Pyridoxamine 5'-phosphate oxidase family protein |
| GE000576 | -1 29916 | 1 13E-05 | WP_153843999 1 | Histone deacetylase family protein |
| GE001961 | -1 12902 | 1 22E-05 | PHS59668 1 | Heme peroxidase |
| GE000216 | 1 103252 | 1 34E-05 | WP_139527888 1 | Metal ABC transporter permease |
| GE003414 | -1 69651 | 1 45E-05 | WP_027965783 1 | Heavy metal translocating P-type atpase |
| GE000261 | 1 180732 | 1 51E-05 | WP_044628772 1 | Oxidoreductase |
| GE000021 | -1 08027 | 1 99E-05 | WP_139526191 1 | Peld GGDEF domain-containing protein |
| GE000023 | -1 30621 | 1 99E-05 | TNH19010 1 | Tetratricopeptide repeat protein |
| GE001948 | -3 00796 | 2 84E-05 | WP_159342318 1 | Hypothetical protein |
| GE003156 | 1 063837 | 3 58E-05 | WP_139525859 1 | Alkene reductase |
| GE001998 | -1 59358 | 3 79E-05 | HBP79862 1 | Hypothetical protein |
| GE003670 | -1 48587 | 4 58E-05 | WP_139526162 1 | Curli assembly protein csgf |
| GE000758 | -1 04551 | 4 68E-05 | WP_054645171 1 | Lyse family transporter |
| GE002816 | -2 16407 | 5 68E-05 | CAD5253492 1 | Hypothetical protein HALO59_120437 |
| GE000970 | -2 53828 | 5 83E-05 | BCB61592 1 | Tail sheath protein |
| GE003168 | -1 03193 | 1 23E-04 | WP_139525871 1 | Efflux RND transporter periplasmic adaptor subunit |
| GE003671 | -1 28249 | 1 28E-04 | WP_139526163 1 | Csge family curli-type amyloid fiber assembly protein |
| GE003148 | -1 14703 | 1 66E-04 | WP_081137861 1 | 4-aminobutyrate--2-oxoglutarate transaminase |
| GE000972 | -1 58446 | 1 89E-04 | WP_139525410 1 | Hypothetical protein |
| GE003250 | -1 2193 | 2 33E-04 | WP_273192629 1 | Transporter |
| GE000879 | -1 05243 | 2 64E-04 | WP_219863109 1 | MULTISPECIES: glucans biosynthesis glucosyltransferase mdoh |
| GE002010 | -1 52849 | 2 73E-04 | QHD50057 1 | Hypothetical protein CTT34_10330 |
| GE002016 | -1 02389 | 3 12E-04 | MAX51659 1 | Hypothetical protein |
| GE003363 | -1 20592 | 4 10E-04 | WP_139525981 1 | Iclr family transcriptional regulator C-terminal domain-containing protein |
| GE003668 | -1 2504 | 5 93E-04 | WP_153843627 1 | Hypothetical protein |
| GE000989 | -1 17796 | 6 51E-04 | WP_253557538 1 | Phage major capsid protein, P2 family |
| GE001957 | -1 04035 | 8 85E-04 | KTG25428 1 | Hypothetical protein AUR68_01260 |
| GE000555 | -1 09532 | 9 26E-04 | WP_044628915 1 | Preprotein translocase subunit yajc |
| GE000988 | -1 03477 | 0 001038 | WP_253017186 1 | Phage terminase small subunit |

**Table S3.** Fifty-eight annotated metabolites identified in MNB13 cells. Bold, metabolites related to EMP, PPP, and TCA cycle.

|  | Compound | Q1 (Da) | Q3 (Da) | Molecular Weight | Ion mode | Ionization model | Formula | –β-Ala  -1 | –β-Ala  -2 | –β-Ala  -3 | +β-Ala  -1 | +β-Ala  -2 | +β-Ala  -3 | CPD ID | CAS |
| --- | --- | --- | --- | --- | --- | --- | --- | --- | --- | --- | --- | --- | --- | --- | --- |
| Amino acid metabolism | L-Aspartate | 132 | 88 | 133.103 | Negative | [M-H]^-^ | C4H7NO4 | 0.209858189 | 0.217334142 | 0.32040071 | 0.193206559 | 0.152899341 | 0.15092926 | C00049 | 56-84-8 |
|  | Glutamine | 147.069 | 84 | 146.069143 | Positive | [M+H]^+^ | C5H10N2O3 | 0.037630236 | 0.032103167 | 0.024203018 | 0.018801766 | 0.018825938 | 0.010650904 | C00064 | 56-85-9 |
|  | L-Asparagine | 131 | 114 | 132.053493 | Negative | [M-H]^-^ | C4H8N2O3 | N/A | 0.01446085 | N/A | N/A | 0.007161046 | N/A | C00152 | 70-47-3 |
|  | L-Leucine | 132.1 | 86.1 | 131.094629 | Positive | [M+H]^+^ | C6H13NO2 | 0.01194068 | 0.014584176 | 0.016060601 | 0.010978448 | 0.012784018 | 0.025735336 | C00123 | 61-90-5 |
|  | L-Citrulline | 174 | 130.9 | 175.095692 | Negative | [M-H]^-^ | C6H13N3O3 | 0.001643035 | N/A | 0.001140996 | 0.001127607 | N/A | N/A | C00327 | 372-75-8 |
|  | Arginine | 175.1 | 70.06 | 174.111676 | Positive | [M+H]^+^ | C6H14N4O2 | 0.527391788 | 0.516858819 | 0.44148519 | 0.300886156 | 0.264952248 | 0.291530172 | C00062 | 74-79-3 |
|  | Tyrosine | 180 | 163 | 181.0738932 | Negative | [M-H]^-^ | C9H11NO3 | 0.048499022 | 0.051254326 | 0.050970593 | 0.03527975 | 0.029986055 | 0.037122211 | C00082 | 60-18-4 |
|  | Lysine | 147.069 | 84 | 146.105528 | Positive | [M+H]^+^ | C6H14N2O2 | 0.001509108 | 0.001403604 | 0.00106014 | 0.000643268 | 0.000486668 | 0.001110417 | C00047 | 56-87-1 |
|  | L-Glutamic acid | 146 | 102 | 147.053159 | Negative | [M-H]^-^ | C5H9NO4 | 0.098843716 | 0.145885585 | 0.14179533 | 0.136123648 | 0.104249493 | 0.258629564 | C00025 | 56-86-0 |
|  | Threonine | 118.050419 | 74 | 119.058244 | Negative | [M-H]^-^ | C4H9NO3 | 0.065594098 | 0.065267102 | 0.059239594 | 0.035814317 | 0.035764959 | 0.040953178 | C00188 | 72-19-5 |
|  | Argininosuccinic acid | 289.2 | 131.9 | 290.122636 | Negative | [M-H]- | C10H18N4O6 | N/A | N/A | N/A | N/A | N/A | 0.000131233 | C03406 | 2387-71-5 |
| Nucleotide metabolism | Guanosine | 282 | 150 | 283.09167 | Negative | [M-H]^-^ | C10H13N5O5 | 0.0900332292 | 0.0690997917 | 0.435997917 | 0.10390875 | 0.0956523958 | 0.857041667 | C00387 | 118-00-3 |
|  | Cyclic-AMP | 328.1 | 134.2 | 329.052522 | Negative | [M-H]^-^ | C10H12N5O6P | 0.160975 | 0.125166667 | 0.146235417 | 0.117055208 | 0.124085417 | 0.091235625 | C00575 | 60-92-4 |
|  | UDP-GlcNAc | 606.1 | 384.9 | 607.0815695 | Negative | [M-H]^-^ | C17H27N3O17P2 | 8.07395833 | 7.02914583 | 8.22498958 | 11.3409375 | 14.4920833 | 10.8758333 | C00043 | 528-04-1 |
|  | dUMP | 307 | 195 | 308.0409519 | Negative | [M-H]^-^ | C9H13N2O8P | 0.506801042 | 0.502672917 | 0.574204167 | 0.56645 | 0.700261458 | 0.333572917 | C00365 | 964-26-1 |
|  | UMP | 323 | 96.9 | 324.03587 | Negative | [M-H]^-^ | C9H13N2O9P | 10.2377292 | 11.0820833 | 11.40875 | 6.92744792 | 14.3025 | 10.73875 | C00105 | 58-97-9 |
|  | Guanosine diphosphate | 441.9 | 149.9 | 443.024334 | Negative | [M-H]^-^ | C10H15N5O11P2 | 1.73091667 | 1.25626042 | 1.35579167 | 2.13559375 | 2.38614583 | 2.01588542 | C00035 | 146-91-8 |
|  | AMP | 345.9 | 79 | 347.063087 | Negative | [M-H]^-^ | C10H14N5O7P | 277.7375 | 271.666667 | 296.40625 | 203.920833 | 261.705208 | 234.645833 | C00020 | 61-19-8 |
|  | dTMP | 321.1 | 195.2 | 322.208 | Negative | [M-H]^-^ | C10H15N2O8P | 1.98436458 | 2.10157292 | 2.49954167 | 2.91955208 | 2.641125 | 3.1756875 | C00364 | 33430-62-5 |
|  | dCMP | 306 | 78.9 | 307.056939 | Negative | [M-H]^-^ | C9H14N3O7P | 4.29978125 | 4.19926042 | 4.44215625 | 5.50058333 | 4.273625 | 5.76233333 | C00239 | 1032-65-1 |
|  | Uracil | 111.2 | 42 | 112.027278 | Negative | [M-H]^-^ | C4H4N2O2 | 1.04012812 | 1.23810417 | 1.87439583 | 1.47832292 | 2.48605208 | 5.28413542 | C00106 | 66-22-8 |
|  | dAMP | 330 | 194.9 | 331.068172 | Negative | [M-H]^-^ | C10H14N5O6P | 13.1127083 | 12.5885417 | 14.1938542 | 12.53875 | 12.1838542 | 13.5246875 | C00360 | 653-63-4 |
|  | IMP | 346.9 | 78.9 | 348.047103 | Negative | [M-H]^-^ | C10H13N4O8P | 454.465625 | 407.517708 | 214.56875 | 553.655208 | 744.276042 | 268.854167 | C00130 | 131-99-7 |
|  | ADP | 426 | 134 | 427.029419 | Negative | [M-H]^-^ | C10H15N5O10P2 | 1.2271875 | 1.34554167 | 1.30876042 | 1.3815 | 1.45420833 | 1.39926042 | C00008 | 58-64-0 |
|  | Inosine | 267 | 135 | 268.080771 | Negative | [M-H]^-^ | C10H12N4O5 | 0.154423958 | 0.141807292 | 0.0827307292 | 0.179877083 | 0.216675 | 0.328365625 | C00294 | 58-63-9 |
|  | Adenine | 134 | 107 | 135.054495 | Negative | [M-H]^-^ | C5H5N5 | 0.54473125 | 0.561988542 | 0.684653125 | 0.512345833 | 0.534613542 | 0.540161458 | C00147 | 73-24-5 |
|  | Nicotinamide adenine dinucleotide | 662.1 | 540.1 | 663.425 | Negative | [M-H]^-^ | C21H27N7O14P2 | 66708.6458 | 58405.1042 | 70665.9375 | 60060.1042 | 64621.5625 | 65991.0417 | C00003 | 53-84-9 |
|  | Triphosphate guanosine | 521.9 | 424.1 | 592.15 | Negative | [M-H]^-^ | C10H16N5Na3O14P3 | N/A | N/A | 0.523445833 | N/A | 0.246579167 | 0.428475 | C00044 | 36051-31-7 |
| TCA cycle | Fumaric acid | 114.9 | 70.9 | 116.01096 | Negative | [M-H]^-^ | C4H4O4 | 10.7847917 | 8.69746875 | 44.7140625 | 11.6877083 | 7.92738542 | 21.6260417 | C00122 | 110-17-8 |
|  | Malic acid | 133 | 114.8 | 134.0215233 | Negative | [M-H]^-^ | C4H6O5 | N/A | 2.38810417 | 2.12121875 | 2.21330208 | N/A | 3.42845833 | C00149 | 6915-15-7 |
|  | Oxaloacetate | 131 | 98.8 | 132.0058732 | Negative | [M-H]^-^ | C4H4O5 | 41.5228125 | 33.7366667 | 40.5727083 | 16.6140625 | 10.9576042 | 22.3536458 | C00036 | 328-42-7 |
|  | *cis*-Aconitic acid | 173.2 | 129.1 | 174.11 | Negative | [M-H]^-^ | C6H6O6 | 0.151111458 | 0.1787125 | 0.176264583 | 0.0811979167 | 0.0469033333 | 0.108307292 | C00417 | 585-84-2 |
|  | alpha-Ketoglutaric acid | 145.0137 | 101.1 | 146.021525 | Negative | [M-H]^-^ | C5H6O5 | 13.1419792 | 13.1727083 | 21.0485417 | 10.6892708 | 7.59238542 | 16.5741667 | C00026 | 328-50-7 |
|  | Succinic Acid | 117.018785 | 73 | 118.02661 | Negative | [M-H]^-^ | C4H6O4 | 49.2671875 | 54.2979167 | 40.851875 | 56.316875 | 44.83875 | 40.3525 | C00042 | 110-15-6 |
|  | Isocitric acid | 190.8 | 73.2 | 192.027005 | Negative | [M-H]^-^ | C6H8O7 | 0.013781875 | 0.0238897917 | 0.0173604167 | 0.0285255208 | 0.017630625 | 0.0191530208 | C00311 | 320-77-4 |
|  | Citric acid | 191 | 129 | 192.027005 | Negative | [M-H]^-^ | C6H8O7 | N/A | N/A | N/A | 2.8628125 | 2.67011458 | 2.595625 | C00158 | 77-92-9 |
| EMP | Pyruvic acid | 86.8 | 43 | 88.016045 | Negative | [M-H]^-^ | C3H4O3 | 1.83857292 | 1.42017708 | 2.32667708 | 1.47148958 | 1.51642708 | 1.7800625 | C00022 | 127-17-3 |
|  | 3-phosphoglycerate | 184.8 | 96.8 | 186.06 | Negative | [M-H]^-^ | C3H7O7P | 0.850716667 | N/A | N/A | 0.942104167 | 0.729007292 | 0.489022917 | C00597 | 820-11-1 |
|  | 2-Phospho-D-glyceric acid | 184.9 | 78.8 | 185.9929391 | Negative | [M-H]^-^ | C3H7O7P | 0.755476042 | N/A | 1.04780208 | 0.68340625 | 0.774060417 | 0.479966667 | C00631 | 3443-57-0 |
|  | Glyceraldehyde-3-phosphate | 168.8 | 96.9 | 170.058 | Negative | [M-H]^-^ | C3H7O6P | N/A | 0.594315625 | 0.6699125 | N/A | 0.630782292 | N/A | C00661 | 591-57-1 |
|  | D-Fructose-6-phosphate | 259.1 | 96.9 | 260.0297185 | Negative | [M-H]^-^ | C6H13O9P | N/A | N/A | N/A | N/A | N/A | 1.31038542 | C00085 | 26177-86-9 |
| PPP | D-Erythrose 4-phosphate | 199.1 | 96.8 | 200.008592 | Negative | [M-H]^-^ | C4H9O7P | 22.7147917 | 17.4664583 | 12.9319792 | 14.0960417 | 12.4089583 | 10.4695833 | C00279 | 585-18-2 |
|  | Xylulose-5-phosphate | 229.2 | 97 | 230.11 | Negative | [M-H]^-^ | C3H7NO3 | 9.34602083 | 5.81357292 | 4.94338542 | 5.38202083 | 5.99726042 | 6.03491667 | C00231 | 4212-65-1 |
|  | D-Ribose 5-phosphate-disodium | 228.9 | 97 | 274.073 | Negative | [M-H]^-^ | C5H9Na2O8P | 11.1419792 | 7.04665625 | 5.09571875 | 7.52220833 | 7.63303125 | 6.72201042 | C00117 | 18265-46-8 |
|  | D-Ribulose-5-phosphate | 229 | 97 | 230.0191538 | Negative | [M-H]^-^ | C5H11O8P | 8.59077083 | 6.32960417 | 4.65832292 | 6.27184375 | 5.89101042 | 7.41173958 | C00199 | 4151-19-3 |
|  | Sedoheptulose-7-phosphate | 289.3 | 78.9 | 290.0402837 | Negative | [M-H]^-^ | C7H15O10P | 64.2433333 | 61.20125 | 50.4934375 | 52.1534375 | 56.0996875 | 54.9601042 | C05382 | 2646-35-7 |
| Others | Glycerol-3-phosphate | 171 | 79 | 172.013677 | Negative | [M-H]^-^ | C3H9O6P | 238.398958 | 163.963542 | 219.505208 | 223.413542 | 293.233333 | 367.363542 | C00093 | 57-03-4 |
|  | D-Mannose-6-phosphate | 258.8 | 97 | 282.118 | Negative | [M-H]^-^ | C6H12NaO9P | 2.77666667 | 1.25510417 | 1.65911458 | 1.67651042 | 2.59745833 | 2.2340625 | C00636 | 70442-25-0 |
|  | DL-Glyceric Acid | 104.7 | 75.1 | 106.077 | Negative | [M-H]^-^ | C3H6O4 | 9.14760417 | 7.47009375 | 5.75248958 | 6.00130208 | 6.66628125 | 3.556125 | C00258 | 473-81-4 |
|  | Ureidopropionate | 130.9 | 88 | 132.118 | Negative | [M-H]^-^ | C4H8N2O3 | N/A | N/A | N/A | 0.0576497917 | 0.0542317708 | 0.0474232292 | C02642 | 462-88-4 |
|  | Glucuronic acid | 192.8 | 73.1 | 194.139 | Negative | [M-H]^-^ | C6H10O7 | 5.19078125 | 5.12697917 | 3.55734375 | 4.23270833 | 4.89716667 | 3.6389375 | C00191 | 528-16-5 |
|  | Cysteic acid | 168.1 | 80.7 | 169.156 | Negative | [M-H]^-^ | C3H7NO5S | 0.768854167 | 1.02662813 | 0.770578125 | 0.501504167 | 0.492167708 | 0.532086458 | C00506 | 498-40-8 |
|  | Glycolic acid | 75 | 75 | 76.051 | Negative | [M-H]^-^ | C2H4O3 | 2.78902083 | 2.55348958 | 2.00535417 | 1.72872917 | 2.05651042 | 1.67490625 | C00160 | 79-14-1 |
|  | L-2-Hydroxyglutaric acid disodium | 146.8 | 85 | 192.08 | Negative | [M-2Na+H]^-^ | C5H6Na2O5 | N/A | 1.38235417 | 1.97520833 | N/A | N/A | 3.86047917 | C03196 | 63512-50-5 |
|  | Flavin mononucleotide | 455.2 | 78.9 | 456.1046148 | Negative | [M-H]^-^ | C17H21N4O9P | 3.44335417 | 3.21198958 | 2.98047917 | 3.70672917 | 3.63780208 | 3.37855208 | C00061 | 146-17-8 |
|  | Argininosuccinic acid | 289.2 | 131.9 | 290.122636 | Negative | [M-H]^-^ | C10H18N4O6 | N/A | N/A | N/A | N/A | N/A | 0.0323488542 | C03406 | 2387-71-5 |
|  | Itaconic acid | 129.1 | 85.2 | 130.1 | Negative | [M-H]^-^ | C5H6O4 | 0.113791667 | 0.135613542 | 0.120008333 | 0.0629838542 | N/A | 0.091755625 | C00490 | 97-65-4 |
|  | Lactate | 89.2 | 43.1 | 90.031695 | Negative | [M-H]^-^ | C3H6O3 | N/A | N/A | N/A | N/A | 3.70586458 | N/A | C00186 | 50-21-5 |

**
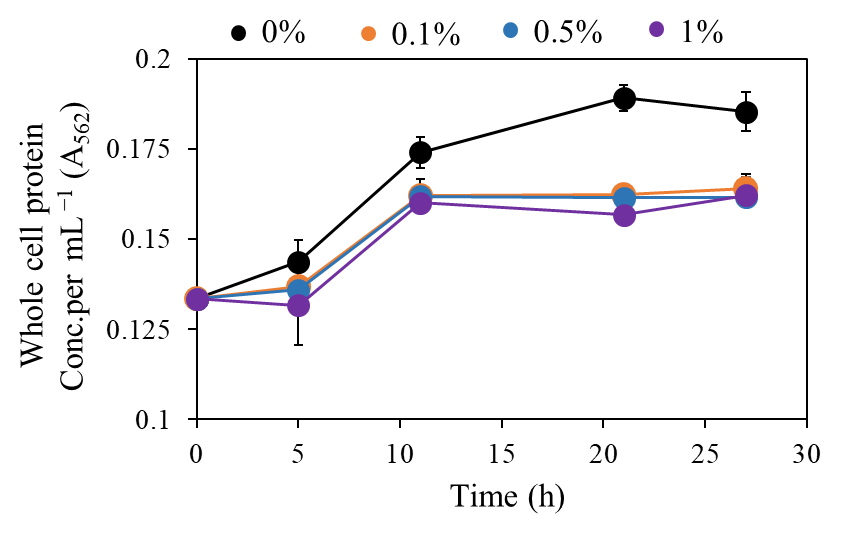
**

**FIG. S1.** Growth curves of strain MNB13 in basal medium with different amounts of MnO_2_ (w/v).


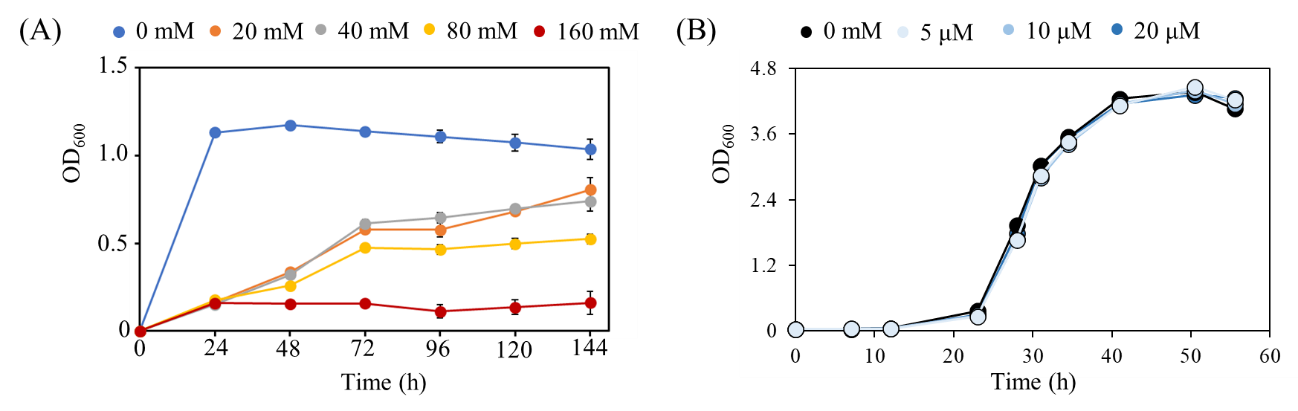


**FIG S2.** Growth curves of strain MNB13 under different concentrations of MnCl_2_: (A) millimolar (mM) range and (B) micromolar (μM) range.

**
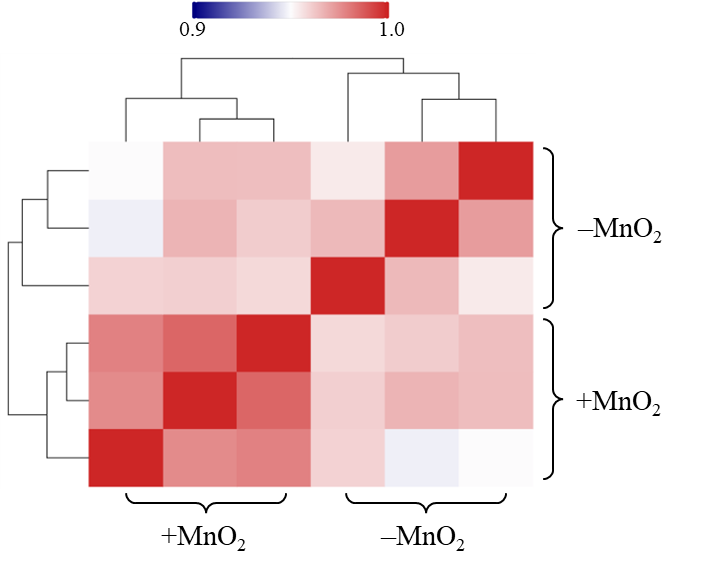
**

**FIG S3.** Gene expression similarity with or without MnO_2_ treatment of *Halomonas* sp. transcriptome. Pearson correlation and dendrogram of hierarchical clustering between all samples in *Halomonas* sp. transcriptome according to gene expression values. Replicates of same treatment show high correlation and cluster together. With MnO_2_ (–MnO_2_) and without MnO_2_ (+MnO_2_) treatments cluster separately.

**
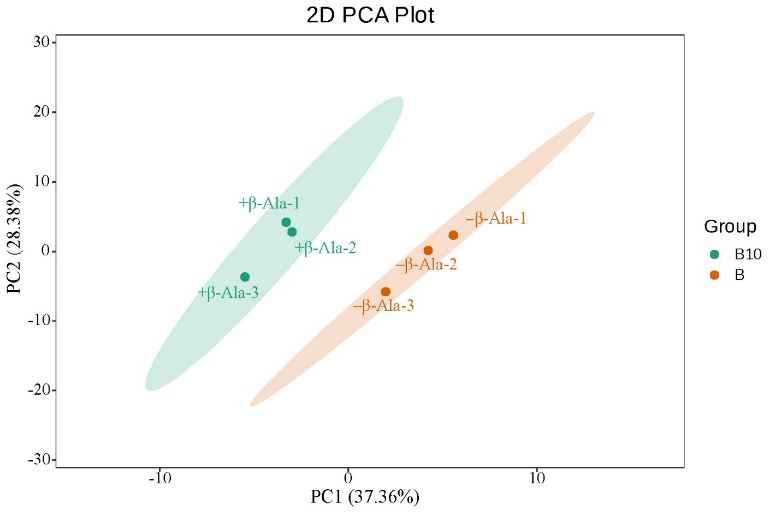
**

**FIG S4.** Principal component analysis of detected metabolites in MNB13 cells with or without β-alanine. Triplicates of each treatment are shown. +, with; –, without.
